# Towards Substitution of Hexane as Extraction Solvent of Food Products and Ingredients with No Regrets

**DOI:** 10.3390/foods11213412

**Published:** 2022-10-28

**Authors:** Christian Cravotto, Anne-Sylvie Fabiano-Tixier, Ombéline Claux, Maryline Abert-Vian, Silvia Tabasso, Giancarlo Cravotto, Farid Chemat

**Affiliations:** 1 GREEN Extraction Team, INRAE, UMR 408, Avignon University, F-84000 Avignon, France; christian.cravotto@alumni.univ-avignon.fr (C.C.); anne-sylvie.fabiano@univ-avignon.fr (A.-S.F.-T.); ombeline.claux@alumni.univ-avignon.fr (O.C.); maryline.vian@univ-avignon.fr (M.A.-V.); 2 Department of Drug Science and Technology, University of Turin, Via P. Giuria 9, 10125 Turin, Italy; silvia.tabasso@unito.it

**Keywords:** hexane, toxicity, 2,5-hexanedione, food industry, alternative extraction methods

## Abstract

Hexane is a solvent used extensively in the food industry for the extraction of various products such as vegetable oils, fats, flavours, fragrances, colour additives or other bioactive ingredients. As it is classified as a “processing aid”, it does not have to be declared on the label under current legislation. Therefore, although traces of hexane may be found in final products, especially in processed products, its presence is not known to consumers. However, hexane, and in particular the *n*-hexane isomer, has been shown to be neurotoxic to humans and has even been listed as a cause of occupational diseases in several European countries since the 1970s. In order to support the European strategy for a toxic-free environment (and toxic-free food), it seemed important to collect scientific information on this substance by reviewing the available literature. This review contains valuable information on the nature and origin of the solvent hexane, its applications in the food industry, its toxicological evaluation and possible alternatives for the extraction of natural products. Numerous publications have investigated the toxicity of hexane, and several studies have demonstrated the presence of its toxic metabolite 2,5-hexanedione (2,5-HD) in the urine of the general, non-occupationally exposed population. Surprisingly, a tolerable daily intake (TDI) has apparently never been established by any food safety authority. Since hexane residues are undoubtedly found in various foods, it seems more than necessary to clearly assess the risks associated with this hidden exposure. A clear indication on food packaging and better information on the toxicity of hexane could encourage the industry to switch towards one of the numerous other alternative extraction methods already developed.

## 1. Introduction

In recent years, consumer behaviour has changed worldwide [1], reflecting growing concerns about health and sustainability following the COVID-19 pandemic. The same trend is also evident in recent policies and particularly in the European Union (EU) with the adoption of the European Green Deal in 2020, which aims to achieve climate neutrality, a circular economy and a toxic-free environment. One of the main keys to achieving these ambitious goals will be the Chemicals Strategy for Sustainability, based on the new Safe and Sustainable-By-Design (SSbD) standard, which aims to ensure that chemicals, materials and products are designed, manufactured and used in a way that does not harm people and the environment [2]. These current priorities are closely related to the research activities carried out by the Department of Chemistry of the University of Turin (Italy) and the GREEN Team of the University of Avignon (France) for several years. One of their common research areas is the use of safe alternative solvents for the production of extracts from biomass instead of potentially hazardous petrochemicals [3,4,5,6,7,8].

Among the solvents used industrially for the extraction of non-polar edible natural products such as colours, flavours, fragrances or lipids, hexane is undoubtedly the most widely used. It is on the list of extraction solvents that may be used in the manufacture of foods or food ingredients in EU Directive 2009/32/ EC [9]. However, this substance is also known to be toxic and was even identified as a cause of occupational diseases in France in 1973. Despite several earlier alarming elements, the toxicity of hexane is still not fully recognised today. At the time of writing, *n*-hexane (the main isomer of hexane) is classified as STOT RE 2 under the regulation on Registration, Evaluation, Authorisation and restriction of Chemicals (REACH), meaning that it is suspected of causing organ damage through prolonged or repeated exposure [10]. Nevertheless, this solvent is currently the focus of European concern, as a process to reclassify *n*-hexane from STOT RE 2 to STOT RE 1 (from suspected to proven neurotoxic to humans) is underway as of 2017 [11]. The legal deadline for the adoption of this new classification is June 2023. According to the latest European Joint Research Centre (JRC) report on Safe and Sustainable by Design Chemicals (SSbD) and materials [12], this category change is expected to have a major impact on the industrial use of hexane, as it will result in a ban on all non-essential uses and a large reduction in plant emissions. In addition, a Regulatory Management Option Analysis (RMOA) for *n*-hexane is currently underway, starting in June 2020. The purpose of this RMOA is to gather information on products containing *n*-hexane and to help authorities decide whether further regulatory risk management measures are needed. In this context, the authors felt it was particularly important to contribute to the collection of scientific information on this substance, which is widely used in the food industry, by writing a review paper. The first part of this work is devoted to the definition of the substance usually referred to as “hexane” with regard to its production and composition. Then, special attention is given to the applications of hexane in the extraction of food products and ingredients, followed by a toxicological evaluation of this substance based on the available literature. Subsequently, various alternatives to hexane for the extraction of natural products are discussed.

## 2. What Is Hexane?

Chemically the name “hexane” refers to a family of molecules with the molecular formula C_6_H_14_. This includes the following five isomers: *n*-hexane, 2-methylpentane, 3-methylpentane, 2,2-dimethylbutane and 2,3-dimethylbutane. However, the composition of technical hexane is not limited to these isomers and varies as it depends on both the petroleum used as feedstock and the refinery’s distillation strategy.

It is important to realise that the term “hexane” can be used to designate various chemical products and could therefore be a source of confusion. In this review, this term refers to the solvent mixture commonly used in industry, usually called “hexanes”, “technical hexane”, or “commercial hexane”. This substance, identified by the EC number 925-292-5 and also designed as “Hydrocarbons, C5-C7, *n*-alkanes, isoalkanes, *n*-hexane rich”, does not have a unique CAS number [13]. As a result, the solvent data sheets provided by suppliers often give a CAS number, which either refers to *n*-hexane (CAS 110-54-3) or to naphtha (petroleum), hydrotreated light (CAS 64742-49-0) and does not allow the product to be accurately identified [14,15,16]. In the following sections, this solvent is defined in terms of its origin, composition and applications.

**Figure 1:**
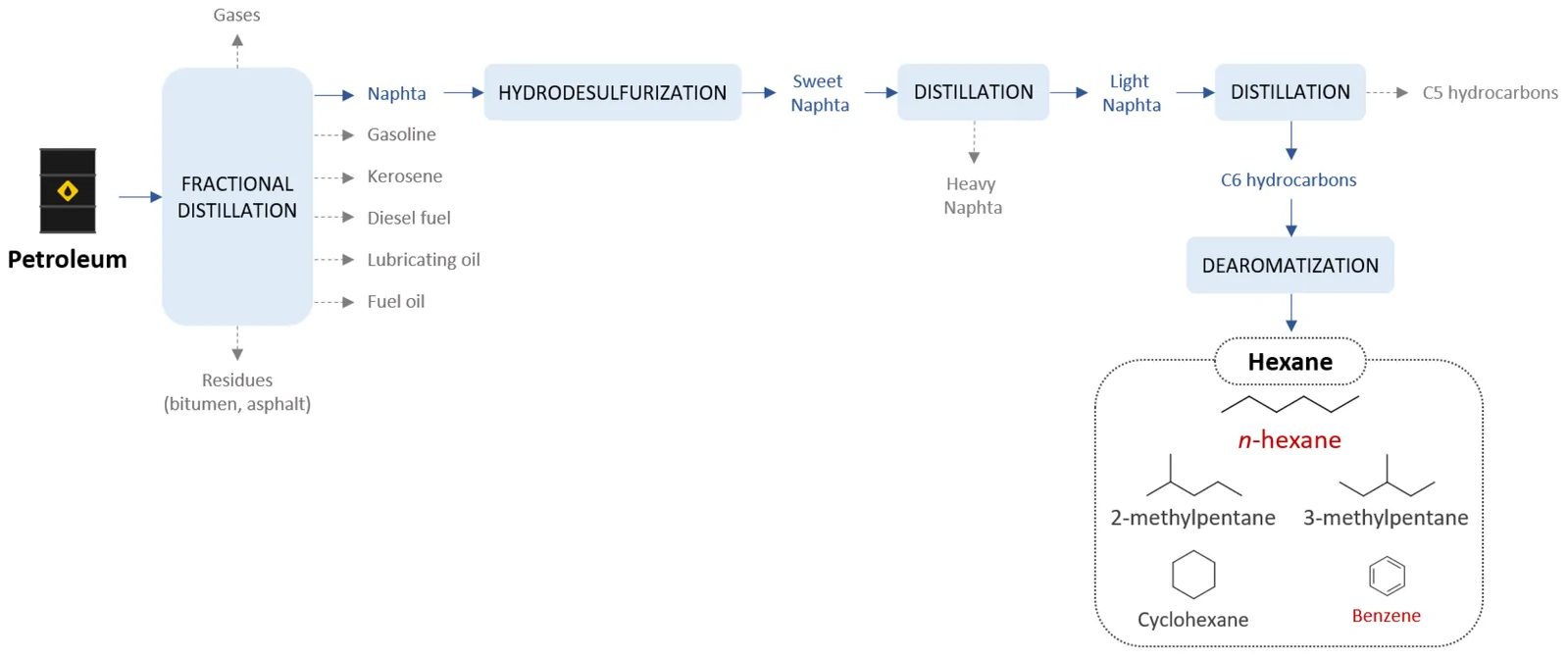
Hexane production and composition.

Hexane is generally produced from naphtha, one of the lightest fractions obtained directly from petroleum refining (see Figure 1). This fraction accounts for about 20% of the weight of crude oil and consists of a mixture of C5 to C10 hydrocarbons with a boiling point in the range of 30–200 °C [17]. The naphtha can then be freed from sulphur and other impurities in a hydrodesulphurisation plant. This purification step is particularly necessary before catalytic reforming of the subsequent cuts to avoid contamination of the metal catalysts [18]. The sweet naphtha obtained is further distilled into heavy naphtha and light naphtha [19]. The former consists mostly of hydrocarbons with more than six carbon atoms and has a boiling point in the range of 140–200 °C. Heavy naphtha is usually fed to a catalytic reformer, and the subsequent reformate is then directly available for blending into gasoline. Light naphtha, on the other hand, usually consists of hydrocarbons with six or fewer carbon atoms and has a boiling point in the range of 30–140 °C. It can be further split into a C5-rich fraction and a C6-rich fraction by additional distillation. Depending on the desired purity, this last fraction is then subjected to dearomatisation to remove aromatic impurities such as benzene [20].

It is important to mention that it is quite difficult to define the exact hexane production process, as each refinery may have its own processing strategy. Therefore, the process shown in Figure 1, which corresponds to the description of Super Petrochemical Limited [21,22], may not be perfectly applicable to every production unit.

The solvent commonly used in the industry has a boiling point in the range of 63–71 °C and consists mainly of *n*-hexane, which usually accounts for 50–85% *v*/*v* of its total content [23]. This linear isomer is known primarily as a neurotoxic and CMR substance (suspected of damaging fertility), which is toxic to aquatic species with long-lasting effects, and as a suspected endocrine disruptor [24,25,26]. Besides this main component, hexane can also contain other hexane isomers, cyclic hydrocarbons such as cyclohexane or even aromatics such as toluene or benzene (see Figure 1). In the 1980s, however, benzene was shown to be carcinogenic and mutagenic, so hexane producers began to refine the hydrocarbon mixture by adding a dearomatisation step to reduce the content of aromatic impurities in the solvent [27].

As a result, depending on the final purity of the product, different grades of hexane are usually offered, e.g., a “food grade”, also called “extraction grade” or “technical grade”, used for the extraction of vegetable oil. Even though there are several standards for the benzene content of “food-grade” hexane (see Table 1), they are not currently applied worldwide, and solvent specifications depend on suppliers. As far as these authors know, there are no regulations for toluene residues.

**Table 1:** Examples of benzene limits recommended in “food grade” hexane.

Organisation	Maximum Benzene Level (mg/kg)	Reference
Bureau of Indian Standards	3300	[27]
ASTM International	1000	[28]
World Health Organization	500	[27]

According to recent estimates [29], about 2 Mt of hexane is produced annually. It can be used for a variety of applications, such as during the production of pharmaceuticals, in polymerisation, in industrial cleaning and degreasing, in the production of adhesives and sealants or in rubber processing, but the most important industrial use is the production of food ingredients and especially oil extraction from oilseeds. For the latter application, the industry needs slightly more than 1 Mt of hexane annually, but this quantity only corresponds to the amount of solvent required to compensate for solvent losses during processing. According to the Best Available Techniques Reference Document for the Food Industry (BAT) [30], two-thirds of these solvent losses are released into the atmosphere, and one-third remain in the food chain, which corresponds to about 350 Kt per year.

## 3. Hexane’s Main Applications in the Extraction of Food Products and Ingredients

**Figure 2:**
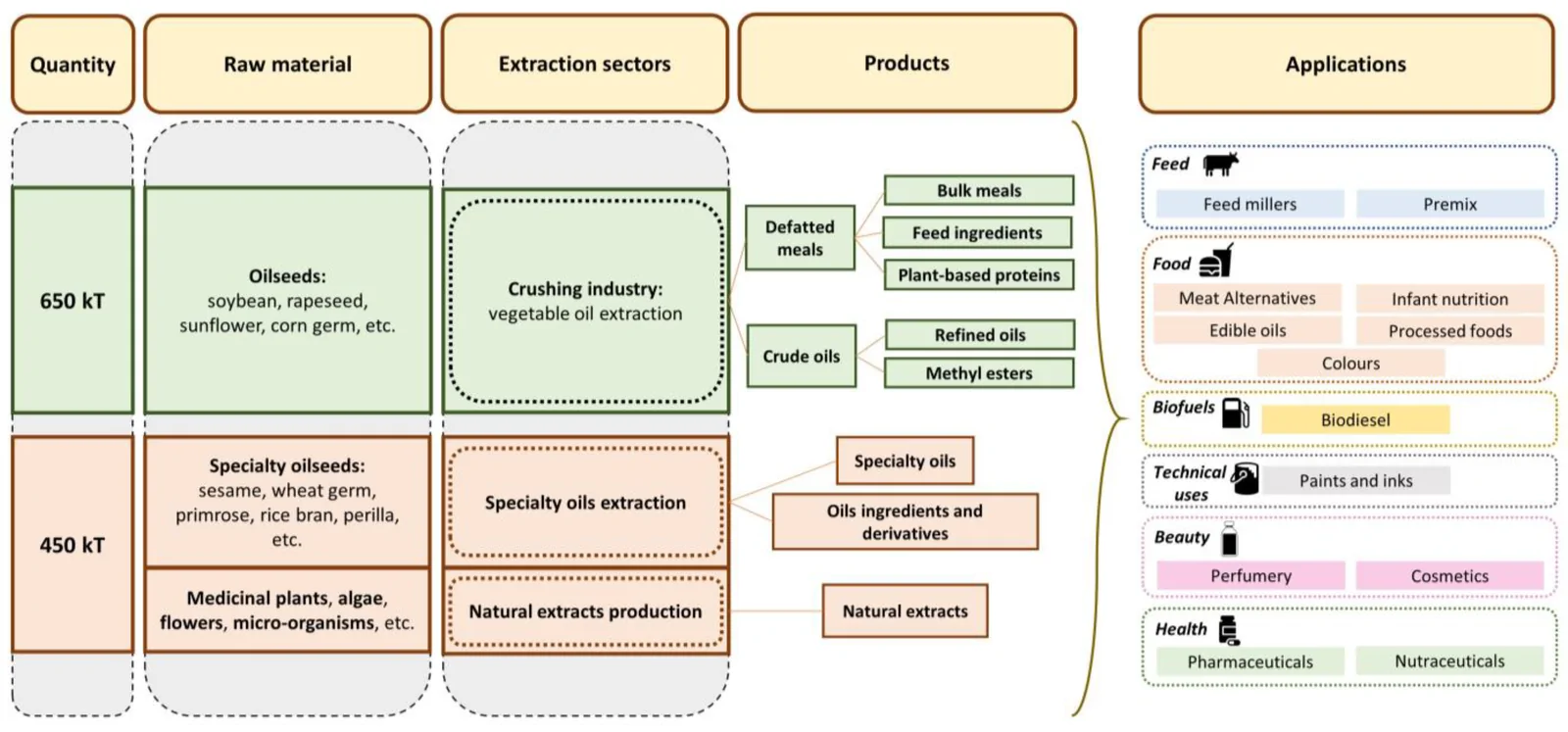
Hexane value chain: products and applications.

Hexane is a petrochemical solvent widely used in the crushing industry for the extraction of vegetable oils but also for the production of flavours and fragrances, natural extracts, drugs and nutraceuticals. The global demand for hexane from these sectors is about 1.1 Mt per year, with 650 Kt used for oilseed extraction and 450 Kt for the production of natural extracts and speciality oils [29] (Figure 2).

In the production of vegetable oil and meal, mechanical pressing and solvent extraction are the two methods commonly used by the industry, the first being the most traditional method of oil extraction [31]. Mechanical pressing is limited to oilseeds with a high oil content due to the low extraction efficiency (about 60–80% of the total oil); sometimes, the press cake, called marc, is fed to a solvent extraction plant to optimise the efficiency of oil extraction, and recover another 5–20% of the oil. For oilseeds with lower oil content, solvent extraction with hexane is the standard practise in modern processing plants. In an oil mill, the seeds are processed into a final product in three steps: preparation of the seeds, extraction of the seeds with hexane, and treatment of the oil and defatted meal. The extraction process consists of a solid-liquid extraction in which the oil is transferred to the solvent by diffusion. After separating the solid, the oil-enriched solvent, called “miscella”, is distilled to remove the solvent and recover the crude oil. The solvent is removed from the defatted solid by desorption and evaporation through direct and indirect steam injection; the wet meal is heated to a temperature high enough to evaporate the solvent without affecting its nutritional value. High protein concentration and quality combined with low anti-nutritional factors are the most desired aspects of the meal. The evaporated solvent is then condensed and recycled in the process. Finally, the crude oil is subjected to chemical or physical refining to remove impurities, contaminants, and solvent residues.

Hexane has become the predominant solvent in oilseeds extraction due to its complete miscibility with oil, low boiling point (about 68 °C), easy recyclability and relatively low cost [32]. This solvent has traditionally been used in the oil industry since the 1930s, and although it is derived from non-renewable resources and is toxic to the environment and human health, it is still widely used in the production of the world’s most consumed seed oils, such as soybean [33], rapeseed [34] and sunflower [35] oils.

In addition, this solvent is used for the extraction of oils with special dietary and functional properties, the so-called speciality oils. These contain significant amounts of desirable components such as antioxidants, essential fatty acids (especially omega-3 and omega-6 fatty acids) and other bioactive compounds such as tocopherols, phytosterols and carotenoids, and are often used as functional foods or in cosmetic products [36]. For some of these oils, such as sesame oil, wheat germ oil, linseed oil, rice bran oil, primrose oil or perilla oil [37,38,39,40], the pressing process is not profitable, as it results in a cake with still a very high oil content. Therefore, solvent extraction is preferred as it affords extraction yields between 98% and 99%. Hexane is also widely used to produce natural extracts (flavours and fragrances, colours, bioactive ingredients, etc.) for the food and beverage, cosmetics, perfumery, or nutraceutical sectors. Flavours and fragrances are complex mixtures of a variety of volatile compounds that are responsible for the organoleptic properties of a product. Two extraction processes are commonly used in the industry to extract aromatic compounds from plants: hydrodistillation and solvent extraction. The first method results in the extraction of an essential oil, while the second in a crude extract called concrete. Hexane is one of the main solvents used to extract volatile compounds from natural sources such as blackcurrant buds [4], caraway seeds [41] and mastic tree leaves [42]. Colour additives are widely used to improve the aesthetic value of food. Natural colours come from renewable resources and can meet consumer expectations for sustainable food. The colours we can see in nature are mainly due to the presence of one or more of the following compounds: anthocyanins, chlorophyll, or carotenoids, which are currently the most important groups of natural colourants used in industry. Due to their hydrophobicity, the extraction of carotenoids is usually carried out with petrochemical organic solvents, especially hexane [43]. Moreover, a large number of natural bioactive ingredients are traditionally extracted from natural sources (plants, sediments, fungi, algae microalgae, etc.) with organic solvents, e.g., hexane can be used for the extraction of non-polar bioactive compounds such as alkaloids, aromatic hydrocarbons, terpenoids, coumarins or fatty acids [44].

## 4. The Toxicological Assessment of Hexane as Extraction Solvent

Hexane was already widely used as a diluent for shoe glues, as a cleaning agent for mechanical parts and as a solvent for food extraction when its human toxicity was reported several decades ago [45]. Toxicity was first associated with neuropathy following inhalation exposure (the main route of exposure in an industrial setting), and the mechanism of action causing the neuronal toxicity is now well described [46]. Although worker exposure to hexane in the industry can be controlled, the toxicity of hexane has never been of public concern, so it is still widely used industrially, especially in the food industry. There is an increasing demand for hexane in all major industries (food, pharmaceutical, chemical, etc.) [29]. This is the result of a general reluctance to abandon this petrochemical solvent and a low perception of the risks associated with its use. Despite the potential exposure from residues of hexane in extracted oils and proteins, the oral toxicity of hexane has not been fully characterised, as confirmed by the lack of an officially recognised Reference Dose for chronic oral exposure (RfD). According to the harmonised classification, *n*-hexane in the EU is an aspiration hazard (Cat. 1), a reproductive toxicity hazard (Cat. 2), an organ toxicity hazard with a repeated or single exposure (Cat. 2 and Cat. 3, respectively), and a skin irritant (Cat. 2). The repeated dose classification Cat. 2 is the classification for neuropathy and has a specific concentration limit of > 5% *w*/*w*, for mixtures containing *n*-hexane [26].

The Integrated Risk Information System (IRIS) is a program of the United States Environmental Protection Agency (U.S. EPA) that aims to identify and characterise the health hazards arising from chemicals present in the environment. IRIS aims to provide toxicity values for the health effects of chronic exposure to chemicals through an oral Reference Dose (RfD), an inhalation Reference Concentration (RfC), and a carcinogenicity assessment. In 2005, U.S. EPA comprehensively reviewed the scientific studies on *n*-hexane toxicity to determine these values [47], which are listed in Table 2. RfC and RfD estimates, respectively, represent the continuous inhalation exposure and the daily oral exposure to the human population (including sensitive subgroups) that are likely to be without an appreciable risk of deleterious effects over a lifetime. The inhalation study on rats by Huang et al. (1989) was selected as the main study for deriving the RfC [48]. From the results in animals, an equivalent concentration for humans was extrapolated and then divided by an uncertainty factor resulting in an RfC value of 0.72 mg/m^3^ in humans. The confidence of the U.S. EPA in the RfC value was medium, reflecting the medium confidence in the main study and in the database, which lacks information on chronic *n*-hexane exposure.

The U.S. EPA concluded that there are no data on the potential oral toxicity of *n*-hexane in humans and that the animal database is limited to two sub-chronic studies unsuitable for calculating an RfD. Furthermore, the toxicity database of *n*-hexane was found to be insufficient for the evaluation of carcinogenic potential in humans [47]. In 2009, the U.S. EPA proposed a provisional RfD value (p-RfD) based on an additional literature review, which, however, did not reveal any new relevant data. A provisional value alone is not sufficient to characterise the adverse effects of a chemical; moreover, the confidence in the proposed p-RfD value of 0.3 mg/kg body weight human/day was low [49]. As far as the authors are aware, no further studies deriving an appropriate oral reference dose have been conducted since 2005, either in Europe or in the United States.

The European Medicines Agency (EMA) document ICH Q3C (R8) on solvent residues recommends acceptable levels of solvent residues in pharmaceutical products for patient safety. The EMA classified *n*-hexane as a Class 2 solvent (solvents to be limited) and proposed a Permitted Daily Exposure (PDE) of 2.9 mg/day, equivalent to 0.058 mg/kg bodyweight/day, assuming a conventional adult human body weight of 50 kg for both sexes [50].

Indicative Occupational Exposure Limits (IOELVs) are intended to ensure the protection of workers’ health at the workplace from the risks posed by hazardous chemicals. According to EC 2006/15, the IOELV for an 8-h exposure to *n*-hexane is 20 ppm or 72 mg/m^3^ [51]. However, not all European countries have adopted the EU’s indicative occupational exposure limit for *n*-hexane.

*n*-Hexane is listed as a cause of occupational disease, at least in France (since 1973), Italy and in Germany. Occupational exposure to *n*-hexane triggers a specific medical follow-up to monitor the exposure and protect the worker.

Humans and animals are potentially exposed to hexane residues when consuming products after extraction. However, legislation on hexane residues in food and feed is not uniform but depends on the area where the food and feed are produced. In Europe, hexane is listed in Directive 2009/32/EC on extraction solvents used in the production of foodstuffs and food ingredients. So far, 20 solvents (7 gaseous and 13 liquid) are listed in the Directive. Hexane is the only product in this list that is not a pure molecule but a variable chemical mixture, defined as follows: “Hexane means a commercial product consisting essentially of acyclic saturated hydrocarbons containing six carbon atoms and distilling between 64 °C and 70 °C”.

According to the European Directive EC 2009/32 [9], the Maximum Residue Limits (MRL) of hexane in extracted foodstuff or food ingredients are 1 mg/kg for oils and foodstuff containing solvent-extracted flavourings and 10 mg/kg in foods containing defatted protein products and defatted flours. In many other countries, food regulations are less restrictive, and often, MRL in food and feed products are not specified, as in the case of the U.S. FDA regulation. Moreover, as far as the authors are aware, there is no specific authority that controls hexane residues in imported products, which poses a problem in limiting consumer exposure in a given area.

According to the European Regulation EU 2013/68 [52] on animal feed materials, hexane is considered a chemical impurity with an allowed maximum concentration of 1000 mg/kg in the final product.

**Table 2:** Hexane reference limits and toxicological evaluation.

**Toxicological Assessment**	**Toxicity Indicators**	**Codes and Values**	**References**
ECHA registration dossier ^a^	Hazard statements	H225, H304, H315, H336, H361f, H373, H411	[26]
Reference concentration for chronic inhalation exposure (general population) ^a^	RfC (mg/m^3^)	0.72	[47]
Reference dose for chronic oral exposure (general population, oral, pharmaceutical residue) ^a^	RfD (mg/kg body weight human/day)	Not assessed due to lack of data	[47]
Permitted daily exposure (general population) ^a^	PDE (mg/day for an average human of 50 kg)	2.9	[50]
Indicative occupational exposure limit values ^a^	IOELV for 8 h of exposure (mg/m^3^)	72	[51]
Maximum residue limits in the extracted foodstuff or food ingredient ^b^	MRL in fat or oil or cocoa butter (mg/kg)	1	[9]
	MRL in food containing defatted protein and defatted flours (mg/kg)	10	
	MRL in defatted soya products as sold to the final consumer (mg/kg)	30	
	MRL in foodstuff containing solvent-extracted flavourings from natural flavouring materials (mg/kg)	1	
Maximum residue limit in animal feed materials ^b^	MRL in feed materials (mg/kg)	1000	[52]

^a^ *n*-hexane (CAS 110-54-3); ^b^ hexane (EC number 925-292-5).

### 4.1. n-Hexane Metabolic Pattern, Excretion, and Toxicity

*n*-Hexane is a colourless, highly volatile liquid that has been shown to have adverse effects on human health. Due to its high volatility, the main route of exposure to *n*-hexane is inhalation, followed by oral exposure and, to a lesser extent, skin contact. Absorption of this solvent has been documented for all three modes of exposure, and in humans, a retention rate of *n*-hexane from inhaled air of 20 to 25% has been described, with the absorption rate increasing with increasing physical activity [53].

The *n*-hexane absorbed into the bloodstream is transported to the liver, which is the most important metabolic organ. As a highly lipophilic solvent, with an octanol/water partition coefficient (log P_o/w_) of 4.00, *n*-hexane is preferentially distributed in the adipose tissue, followed by the liver, brain, muscles, kidneys, heart, and lungs, respectively [47]. Veulemans et al. (1982) studied the time course of *n*-hexane concentration in blood circulation, which reaches a plateau in 100 min after inhalation exposure and has an average half-life in the blood of 1.5–2 h [54]. In addition, Bus et al. (1979) recorded placental barrier passage of the solvent in pregnant rats exposed by inhalation and demonstrated that the concentrations of *n*-hexane and two of its metabolites (methyl *n*-butyl ketone and 2,5-hexanedione) in the foetus and maternal blood were similar over time [55].

**Figure 3:**
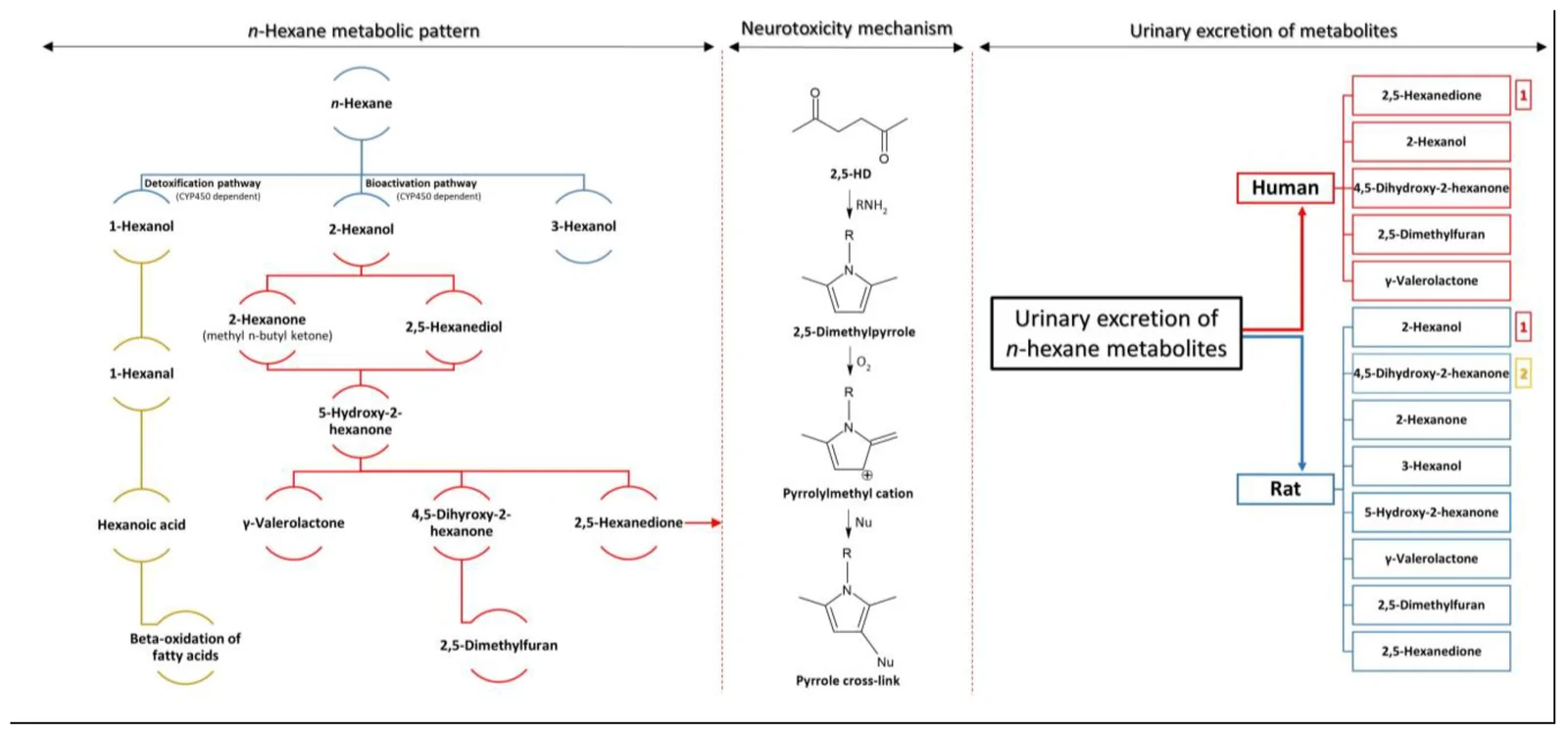
*n*-Hexane metabolic pattern, neurotoxicity mechanism, and metabolites urinary excretion.

*n*-Hexane toxicity is mainly due to its major human metabolite 2,5-hexanedione (2,5-HD), the γ-diketone metabolite of both *n*-hexane and methyl *n*-butyl ketone (MBK). The concentration of 2,5-HD in urine is usually determined by gas chromatographic analysis and used as an indicator of *n*-hexane exposure [56]. The total 2,5-HD content is measured after acid hydrolysis of the urine sample, whereas the free 2,5-HD amount is determined without initial acidification. Although total 2,5-HD correlates more strongly with *n*-hexane exposure [56], Fedtke and Bolt (1986) suggested that the total content may overestimate the 2,5-HD concentration due to the additional production of this compound from 4,5-dihydroxy-2-hexanone, a detoxification metabolite of *n*-hexane [57]. A small portion of the absorbed *n*-hexane is excreted in the exhaled air in an unmetabolised form (up to 10%), while the rest is excreted in the form of metabolites in the urine. While 2,5-HD is the main metabolite found in human urine samples after hexane exposure, Fedtke and Bolt (1987) identified 2-hexanol as the one mainly present in rat urine, which, together with 4,5-dihydroxy-2-hexanone, accounts for approximately 90% of the total excretion during the first 8 h after inhalation exposure [58]. The metabolic pattern of *n*-hexane, the neurotoxicity mechanism and its metabolites in urine are shown in Figure 3.

The nervous system is the main target of *n*-hexane toxicity, and its neurotoxic potential was first documented in occupationally exposed humans and then confirmed in experimental animals. The neurotoxic properties are related to the *n*-hexane metabolite 2,5-HD, endowed with a γ-diketone structure responsible for the toxic effect. The gamma spacing between the two carbonyl groups in 2,5-HD is crucial for the neurotoxicity onset, which is why the term “γ-diketone neuropathy” is often used [46]. Krasavage et al. (1980) confirmed that the magnitude of the neurotoxic effect of *n*-hexane in rats was directly related to the amount of 2,5-HD produced, ranking the relative neurotoxicity of its metabolites in the following descending order: 2,5-HD, 5-hydroxy-2-hexanone, 2,5-hexanediol, 2-hexanone, 2-hexanol and *n*-hexane, after direct oral administration of each metabolite to male rats (5 animals per group) for 17 to 100 days. The endpoint for neuropathy assessment was severe weakness or paralysis of the hind limbs as evidenced by the “dragging” of at least one hind foot. Within 20 days, all rats given 2,5-HD at a dose of 755 mg/kg body weight/day reached the endpoint, whereas it took more than 82 days for rats given 2-hexanol at a dose of 675 mg/kg body weight/day. By determining the relative toxicity of the metabolites, the authors suggested a neurotoxic potency of 2,5-HD, which is 3.3 times that of MBK and 38 times that of *n*-hexane [59].

After cyclisation, 2,5-HD carbonyl groups first react with the amine functions of proteins (ε-amino group of lysine) to produce pyrrole adducts. These derivatives are then oxidised to electrophilic oxidised pyrrole rings, which finally react with other nucleophilic groups of protein, resulting in crosslinked proteins [46]. In axons, this process results in neurofilament cross-linking, which leads to multifocal axonal swelling [60,61].

The distal portion of long, large-diameter peripheral axons is the main target of *n*-hexane toxicity, leading to a reduction of nerve conduction velocity caused by distal axonal degeneration, reduction of axonal transport, and focal demyelination. Recently, LoPachin and Gavin (2015) proposed axonal atrophy as a likely causal factor for peripheral nerve dysfunction, as it was a predominant effect that occurred regardless of the 2,5-HD dose [62].

Peripheral nerve biopsies from human patients exposed to *n*-hexane confirmed the presence of paranodal axonal swelling, myelin retraction from the nodes of Ranvier, and distal axonal degeneration [46]. In addition, small-diameter nerve fibers and unmyelinated nerve fibers also show degenerative changes, although they are less susceptible to the toxicity of *n*-hexane. Despite the low acute toxicity of this solvent, exposure to high *n*-hexane concentrations (above 1000 ppm) causes irritation of the eyes and mucous membranes of the respiratory tract, as well as central nervous system depression leading to an initial euphoric state followed by drowsiness with headache, dizziness, and nausea. Furthermore, the neurological toxicity of this solvent may be exacerbated by concurrent exposure to other substances, especially methyl ethyl ketone [53].

**Figure 4:**
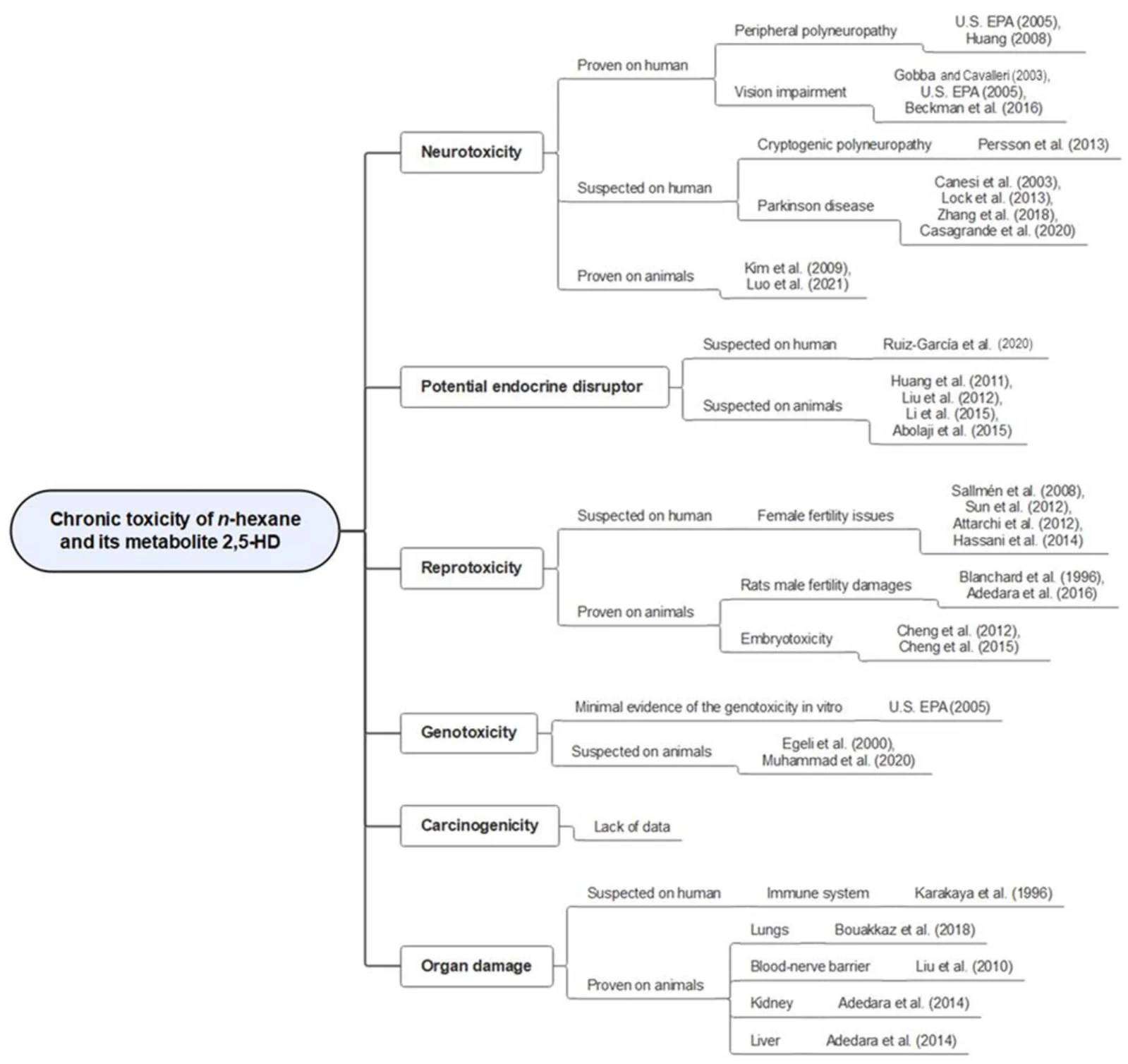
Chronic toxicity of *n*-hexane and its metabolite 2,5-HD [24,25,47,63,64,65,66,67,68,69,70,71,72,73,74,75,76,77,78,79,80,81,82,83,84,85,86,87,88,89].

After prolonged exposure to *n*-hexane over a period of months, neurotoxicity initially manifests as symptoms of paraesthesia (numbness and reduced sensitivity) in the feet and hands, followed by loss of distal reflexes and weakness of intrinsic muscles in the legs and arms, leading to peripheral sensory polyneuritis. With continued exposure, there is a proximal progression of loss of sensory and motor function, leading to symmetric and distal sensorimotor neuropathy. The chronic toxicity of *n*-hexane and its metabolite 2,5-HD is shown in Figure 4.

The 2005 U.S. EPA report reviewed scientific studies on the toxicity of *n*-hexane in occupationally exposed persons (e.g., workers in shoe factories and printing plants). In addition, Huang (2008) reviewed the outbreaks of polyneuropathies associated with occupational exposure to *n*-hexane in Taiwan [47,63]. These reviews establish a strong association between long-term exposure to *n*-hexane and the occurrence of peripheral neuropathies. However, in some studies, workers were exposed to mixtures of volatile solvents, and the proportion of exposure to *n*-hexane alone was not reported. After cessation of exposure, it was observed that the severity of the neurological deficit increased within 1 to 4 months, with deterioration of muscle strength and sensory deficits being maximal. Symptoms disappeared only slowly, and long-term studies indicated that while motor nerve function recovers more quickly, sensory nerves are more severely affected and may not fully recover until ten years after exposure.

Workers occupationally exposed to various solvents, including *n*-hexane, have been shown to have colour vision disorders. These disorders usually result in a loss of blue-yellow colour discrimination and, less commonly, a combination of blue-yellow and red-green colour loss. A possible cause of vision disorders induced by *n*-hexane could be a distal axonopathy of the optic pathway, but the exact pathogenesis is still unclear. The major industrial solvents associated with colour vision disorders were reviewed by Gobba and Cavalleri (2003), while the U.S. EPA (2005) reported three studies documenting maculopathy and colour vision disorders in individuals exposed to *n*-hexane. In addition, in one investigative study, colour vision disturbances were distributed across the spectrum of colour vision deficits [47,64]. Beckman et al. (2016) examined the effects of exposure to hexane and non-hexane solvents on colour vision in 835 automotive repair workers. Although no statistically significant association was found between blue-yellow defects and hexane exposure in younger participants, prevalence rates were higher in the most exposed participants. The long-time interval between cessation of *n*-hexane exposure and colour vision assessment (5–13 years) may have attenuated the observed associations. Furthermore, it has been suggested that concurrent exposure to acetone may potentiate the toxic effects of *n*-hexane on vision [65].

The incidence rate of polyneuropathy ranges from 1 to 3% in the general population and reaches 7% in the elderly. Although more than 100 different causes of polyneuropathies have been identified, and even with strict application of diagnostic guidelines, approximately 20–30% of polyneuropathies have no identifiable cause, resulting in cryptogenic polyneuropathy [90]. Cryptogenic polyneuropathy usually begins in the sixth decade of life, with an insidious onset of symptoms and a higher incidence in men. Persson et al. (2013) measured 2,5-HD concentrations in urine samples after acid hydrolysis in 114 cases of cryptogenic polyneuropathy (0.48 mg/L), comparing them with 227 reference subjects (0.41 mg/L) and concluded that the former cases had significantly higher urinary 2,5-HD concentrations. In both groups, no one had been occupationally exposed to *n*-hexane or MBK. There was no difference between 2,5-HD levels in smokers and non-smokers, while excretion was higher in men than in women and decreased with age in both sexes. The study suggested that external exposure may partially explain the higher 2,5-HD levels, but the small difference in 2,5-HD mean values between the cases studied and the general population did not allow a clear determination of the causality of polyneuropathy [66].

Neurological diseases are the main cause of disability worldwide, and among these, Parkinson’s disease (PD) is the disease with the highest growth rate of cases. In the last 30 years, the number of people with PD has doubled to more than 6 million, and it is expected to double further by 2040 [91]. PD is a debilitating neurodegenerative disease characterised mainly by motor symptoms (resting tremor, bradykinesia, postural instability, and gait disturbance) and is largely due to the loss of dopaminergic neurons in the substantia nigra pars compacta. Although several genetic mutations have been found in PD patients, about 90% of PD cases occur sporadically, and environmental factors play a key role in the development of this disease. The causes of PD are still partly unclear, but certain toxic agents, including *n*-hexane, may play an aetiological role.

Pezzoli et al. (2000) studied the effects of exposure to some hydrocarbon solvents in PD patients and showed that exposed patients had earlier disease onset, more severe disease progression, and poorer response to treatment, suggesting that some hydrocarbon solvents may be involved in the aetiopathogenesis of PD [92]. However, the results were not reported for exposure to single solvents. Lock et al. (2013) reviewed the toxicological and epidemiological evidence for the association between solvent exposure and PD. Although cases of parkinsonism have been associated with exposure to various solvents, including *n*-hexane, there is currently no clear evidence that any particular solvent or class of solvents is an established cause of PD, and the authors concluded that further studies are therefore needed [67]. Recently, Zhang et al. (2018) found progressive dopaminergic neurodegeneration in the nigrostriatal system of mice exposed to 2,5-HD, the toxic metabolite of *n*-hexane. The cause of the loss of both nigral dopaminergic neurons and striatal dopaminergic terminals was microglial activation mediated by the integrin oxidase (α_M_β_2_-NOX2) axis. The authors suggested that these results provide experimental evidence for the contribution of *n*-hexane exposure to the aetiology of PD [68]. Furthermore, microtubule dysfunction has been suggested as a possible crucial event triggering neuronal degeneration in PD. In the study by Casagrande et al. (2020), impairment of the cell’s microtubule system was shown to be an early event in the 2,5-HD-induced neurodegeneration of the dopaminergic system, suggesting that this event may play a key role in the neuronal death process [69]. Poor elimination of *n*-hexane has also been implicated as a risk factor in PD; indeed, the study by Canesi et al. (2003) reported that for a similar quantity of *n*-hexane in the blood, the urinary levels of 2,5-HD were significantly lower in the 108 patients with PD than in healthy controls [70].

Although experimental studies have used various animal models to describe the mechanisms of peripheral neuropathy, *n*-hexane neuropathy has been most thoroughly studied in rats [93]. The neurotoxicity of this solvent has been extensively described in animal models, through exposure to *n*-hexane itself or its metabolite 2,5-HD, mainly through inhalation exposure, while few studies have investigated the toxic effects following oral exposure [47]. Neuronal apoptosis has been proposed as a key factor contributing to *n*-hexane-induced neurological dysfunction. In 2021, Luo et al. confirmed that 2,5-HD induces pathological apoptotic responses both in vitro (VSC4.1 cells) and in vivo (rat spinal cord neurons). The authors, investigating the toxic mechanism of action, suggested that 2,5-HD increased the concentration of pro-nerve growth factor (proNGF) by reducing its conversion to mature nerve growth factor (mNGF) and upregulating a signalling pathway that eventually leads to neuronal apoptosis [71]. In addition, Kim et al. (2009) investigated the toxic effects of low doses of 2,5-HD on hippocampal neurogenesis in the central nervous system. Adult neurogenesis is a striking form of neuronal plasticity that occurs in specific brain regions, including the hippocampus, in several mammalian species. Although direct evidence for adult neurogenesis in humans remains elusive, some studies support its existence, and the marked impairment of this process in neurodegenerative diseases (such as Alzheimer’s disease) supports this evidence [94]. In the study by Kim et al. (2009), micromolar concentrations of 2,5-HD suppressed the proliferation and viability of neural progenitor cells (from 500 nM to 50 µM) and impaired neurogenesis in the hippocampus of adult mice at very low doses (10 or 50 mg/kg), possibly through increased formation of reactive oxygen species (ROS) and microglial activation [72].

In the last two decades, the increased interest in endocrine disruption in humans and the laboratory detection of chemicals with endocrine-disrupting properties associated with pathological outcomes have drawn attention to a heterogeneous class of chemicals that have the ability to alter various mechanisms of the endocrine system and are referred to as endocrine disruptors [95].

Ruiz-García et al. (2020) investigated the possible role of *n*-hexane as an endocrine disruptor in occupationally exposed women of reproductive age working in a leather shoe factory [25]. *n*-Hexane was the solvent with the highest concentration (mean 49.7 mg/m^3^) among the seven solvents measured at the workplace, and the 2,5-HD urine levels were statistically higher (*p* < 0.001) in the group of female workers exposed to solvents (224.4 μg/L post-shift sample, *n* = 34), compared to a non-exposed control group (42.8 μg/L, *n* = 32). To assess a possible association between exposure to *n*-hexane and alterations in ovarian function and the hypothalamic-pituitary-ovarian axis, levels of several hormones were determined in both groups. In the exposed group, oligomenorrhoea was more frequent and time to pregnancy was longer than in the control group. The authors found no significant differences in serum levels of ovarian hormones and gonadotropins in the exposed group compared to controls and classified the disorder as anovulatory dysfunction. However, significant associations were observed between serum FSH levels and urine 2,5-HD concentrations in non-smoking participants suffering from oligomenorrhoea in the exposed group (*n* = 23). The authors concluded that the reduction of FSH by 2,5-HD caused increased apoptosis in growing follicles in the ovaries of the exposed women and suggested that *n*-hexane is an endocrine disruptor in women of reproductive age. However, they recommend that these preliminary results should be confirmed in a larger population and that it is necessary to clarify whether metabolites of volatile organic compounds (VOCs) other than 2,5-HD are involved in the endocrine effects. In addition, several studies have shown that *n*-hexane can disrupt the female reproductive hormone system. For example, using the same protocol of inhalation exposure to *n*-hexane, Huang et al. (2011) and Liu et al. (2012) demonstrated that *n*-hexane has an apparent disruptive function on the reproductive hormone system in mice, particularly on progesterone levels [73,74]. Although serum levels of the ovulating hormone (LH), the follicle-stimulating hormone (FSH) and oestradiol did not change significantly in the in vivo tests, progesterone levels decreased significantly in all groups exposed to *n*-hexane compared to the control.

In in vivo and in vitro experiments with ovarian culture medium, oestradiol and progesterone levels decreased significantly after exposure to *n*-hexane or 2,5-HD. Moreover, after exposure to *n*-hexane, the ovarian response (superovulation) to excessive gonadotropin levels decreased markedly. In general, mice exposed to *n*-hexane showed an abnormal oestrus cycle, with a significant shortening of the duration of the diestrus stage in the group with the highest exposure (75.8 mL/m^3^). Furthermore, *n*-hexane exposure inhibited follicular development and promoted follicular atresia and luteal degeneration, and the results suggested that follicular dysplasia may be due to ovarian granulosa cell apoptosis.

It is unclear whether the disruption of hormone levels is due to *n*-hexane-induced damage to the gonads or whether it is secondary to hormonal alterations. As Abolaji et al. (2015), show, 2,5-HD-induced ovarian and uterine oxidative stress and altered the endocrine balance in rats. It also showed a pronounced effect on the ovaries, where, in contrast to the uterus, there was a significant decrease in the activities of the four major antioxidant enzymes, suggesting a possible impairment of ovarian function and integrity. However, in contrast to other studies, there was no decrease in progesterone levels but a significant increase in FSH and LH levels, while a decrease in oestrogen was noted [24]. The authors suggested that 2,5-HD may have a direct negative effect on the ovaries, leading to an alteration of the normal feedback mechanism and resulting in elevated LH and FSH levels.

In addition, endocrine disruption and abnormal oestrus cycles were observed in the offspring of adult female rats following inhalation of 100, 500, 2500 or 12,500 ppm of *n*-hexane during gestation. In the study by Li et al. (2015), this resulted in the disruption of oestrus cycles, follicular development disorders and altered hormone secretion by ovarian granulosa cells in the offspring. These events have been associated with alterations in the expression level and methylation status of key hormone production genes by ovarian granulosa cells due to the possible disruption of de novo methylation of genes in embryos during gestation and the maintenance of this disruption until birth [75].

Numerous human studies have confirmed that occupational exposure to organic solvents is associated with menstrual disorders and reduced fertility and has adverse effects on pregnancy, including an increased risk of spontaneous abortion. Nevertheless, it is difficult to extrapolate the toxic effects of individual substances due to exposure to solvent mixtures. We selected three retrospective studies investigating the effects on fertility in women of reproductive age occupationally exposed to organic solvents. In all studies, the identification and quantification of organic solvents (including *n*-hexane) were performed according to industrial hygiene methodology, and workers were divided into groups based on exposure intensity. In the study by Sallmén et al. (2008), exposure of workers in shoe manufacturing to solvents was associated with reduced fertility, expressed as an adjusted fecundability density ratio (FDR). Furthermore, no conventional dose-response relationship was found, with effects being more pronounced for exposures at lower concentrations and a stronger association for short-term than for longer-term exposures [76]. In the study by Attarchi et al. (2012), the concentrations of the organic solvent mixtures were within the threshold limit, with the main solvents being formaldehyde, phenol, *n*-hexane and chloroform, with average concentrations of 0.01, 0.5, 20.7 and 3.2 ppm, respectively [77]. Exposure to the same compounds and at the same concentrations was reported in the study by Hassani et al. (2014) for the low-exposure group, while in the high-exposure group, the concentrations were 0.03, 12, 35, and 5.1 ppm, respectively [78]. In the study by Attarchi et al. (2012), a significant association between occupational exposure to mixed organic solvents and increased time to pregnancy was observed, with the dose-response relationship described by odds ratios that increased from 2.76 in the low-exposure group to 4.48 in the high-exposure group. Furthermore, the spontaneous abortion rate was significantly higher in women with occupational exposure to organic solvent mixtures, both at high and low concentrations (*p* < 0.05). Hassani et al. (2014) reported odds ratios for the prevalence of menstrual disorders of 3.40 (*p* = 0.002) in the low-exposure group and 9.69 (*p* = 0.001) in the high-exposure group compared to the control, with mean cycle length, duration, and amount of bleeding, and prevalence of dysmenorrhoea being significantly higher in the exposed groups (*p* ≤ 0.05). In addition, the mean values of FSH, LH and thyroid stimulating hormone (TSH) were significantly higher in the exposed groups, confirming the presence of hormonal changes following exposure to organic solvents, whereas oestrogen and progesterone levels remained unaffected.

Although the negative effects of exposure to mixtures of organic solvents on female fertility are well established, it is difficult to identify the individual causal agents. Laboratory studies, as the work by Sun et al. (2012), made it possible to evaluate the effects of a single toxic agent on human reproductive cells. In this study, human ovarian granulosa cells were exposed to micromolar concentrations of 2,5-HD in vitro for 24 h, showing that 2,5-HD caused cell apoptosis in a dose-dependent way. When the apoptotic mechanism was examined, a decrease in the expression of BCL-2 (important anti-apoptotic protein) and an induction of the expression of BAX (BCL-2 antagonist protein) and CASPASE-3 (key protease in the apoptotic process) were observed [79].

In addition to its toxicity to the female reproductive system, *n*-hexane also has negative effects on the male sex, especially on Sertoli cells. These cells play a key role in the proliferation and differentiation of germ cells in the seminiferous epithelium of the testis. In Sertoli cells, 2,5-HD also disrupts various inter- and extracellular systems by causing the appearance of numerous large vacuoles, altering adhesion complexes at junctions. It also disrupts the kinetics of microtubule assembly and disassembly, leading to the disruption of vesicle transport along filamentous microtubule networks [96]. Blanchard et al. (1996) reported a substantial increase in germ cell apoptosis of the seminiferous epithelium of rats treated with 1% (*v*/*v*) 2,5-HD in drinking water for five weeks. The intensity of germ cell apoptosis increased after only two weeks of exposure and peaked after five weeks, resulting in germ cell loss and testicular atrophy in the rat. Apoptosis was suggested as the mechanism of testicular damage induced by 2,5-HD. Moreover, no rat showed signs of spermatogenesis until 24 weeks after exposure, resulting in irreversible testicular atrophy [80].

In addition, 2,5-HD also causes the induction of oxidative stress in the testicular tissue of rats, as shown by Adedara et al. (2016). Rats in the most exposed group (1% 2,5-HD in drinking water) showed a significant decrease in body weight, testicular weight, and epididymal weight compared to the control group. Increased levels of hydrogen peroxide (H_2_O_2_) and malondialdehyde (MDA) in testes and spermatozoa indicated oxidative damage due to the inability of antioxidant enzymes to counteract oxidative induction. The increased oxidative stress induced by 2,5-HD was confirmed by the significant dose-dependent increase in the activities of antioxidant defence mechanisms, including superoxide dismutase (SOD), catalase (CAT), and glutathione peroxidase (GPx) in both testes and spermatozoa, and by the reduction of glutathione (GSH) and glutathione S-transferase (GST). Consistent with other studies, 2,5-HD resulted in testicular and epididymal atrophy with a significant dose-dependent decrease in epididymal sperm count, sperm motility, and sperm viability. In addition, histopathological studies confirmed that oxidative stress caused severe lesions characterised by vacuolization and degeneration of the testicular tubules and erosion of the epididymal lining with reduced integrity of the epithelial layer [81].

*n*-Hexane can cross the placental barrier, and Bus et al. (1979) reported the presence of 2,5-HD in the foetus after maternal exposure in rats [55]. Two studies by Cheng et al. (2012, 2015) investigated the embryotoxic effects (chick embryos) of 2,5-HD exposure in relation to neurodevelopment, angiogenesis, and vasculogenesis [82,83]. Exposure to 2,5-HD resulted in embryonic malformations, with marked effects on the formation of the neural tube (neurulation), the early developmental stage of the central nervous system. It has been shown that in the presence of 2,5-HD, the forebrain, midbrain, and occasionally the boot neural tube develop morphological abnormalities.

Embryo malformation rates increased in a dose-dependent manner and were 60% (10 mM 2,5-HD), 64% (100 mM), and 70% (1000 mM), while death occurred in 14% and 30% of the 100 mM and 1000 mM treated groups, respectively. Treatment with 2,5-HD resulted in decreased expression of neurofilaments and a decreased number of neurons, while neurites arising from neurons were significantly shorter than in control cultures. In addition, 2,5-HD significantly reduced cell viability and increased apoptosis levels in in-vitro assays. Finally, numerous other malformations were observed in the embryos, such as scoliosis, abnormal somites, and cardiovascular development, suggesting that the embryotoxic effect of 2,5-HD may not be specific to the nervous system [82]. In the second study, disorders of vasculogenesis, through altered blood island formation by inducing apoptosis of angiogenic mesenchyme and angiogenesis, through the impaired expression of VEGF-R, FGF-2 and angiogenin, were caused by 2,5-HD exposure. Consistent with other studies [24,72,81], exposure to 2,5-HD increased the production of ROS, which may lead to the occurrence of vascular dysplasia, which has been proposed as a probable cause of extravascular hemolysis and embryonic death [83].

In 2005, the U.S. agency EPA reviewed numerous studies examining the mutagenicity and genotoxicity of *n*-hexane and commercial hexane mixtures both in vitro and in vivo [47]. They concluded that data from short-term in vitro tests provided minimal evidence of the genotoxicity of *n*-hexane and commercial hexane mixtures. Furthermore, in vivo tests on genotoxic potential were predominantly negative, but some studies provided contradictory results. For example, the toxicological dossier on *n*-hexane from the French National Institute for Research and Occupational Safety (INRS, 2019) reported that *n*-hexane causes chromosomal aberrations and sperm abnormalities in rats after inhalation exposure [53]. Egeli et al. (2000) studied genotoxic and hemotoxic effects in rats subcutaneously exposed to hexane (not specifying whether it was *n*-hexane or technical hexane) and compared them with a non-exposed group and a positive control group exposed to benzene [84]. Although the genotoxic effect of benzene was more pronounced, hexane (0.125 and 0.250 mL/kg in a single dose for three months) showed a significant clastogenic effect in rat bone marrow cells, with an increase in chromosomal aberrations compared to unexposed controls. In addition, high concentrations of conjugated dienes and MDA were detected in the hexane-exposed groups, suggesting covalent binding of DNA and resulting DNA damage. Histological examinations revealed intracytoplasmic vacuolization, reduced chromatin content, and parenchymal degeneration in the bone marrow cells. In addition, the concentrations of haematocrit, haemoglobin, and mean corpuscular volume decreased, indicating haemotoxicity, while increased alanine aminotransferase (ALT), aspartate aminotransferase (AST), and catalase (CAT) activity were signs of hepatotoxicity in the hexane-exposed groups. Hepatotoxicity was also confirmed by cellular, histological examination. In conclusion, Egeli et al. (2000) suggested that hexane is a genotoxic and hemotoxic agent leading to chromosome aberrations and lipid peroxidation in the exposed groups [84].

Recently, the study by Muhammad et al. (2020) investigated the genotoxic effects of 2,5-HD in detail by examining both computational molecular docking to DNA and in vivo exposure models in rats. Based on in silico studies, it was shown that 2,5-HD forms a strong hydrophobic interaction with DNA that could potentially lead to DNA fragmentation. Exposure to 2,5-HD significantly (*p* < 0.05) increased DNA fragmentation in all tissues and organs studied (blood, liver, brain, heart, lung, kidney, pancreas) compared to the unexposed group. Furthermore, this effect was associated with a significant increase in protein carbonyl and MDA levels in all organs (except in the liver), a symptom of oxidative stress. 2,5-HD caused chromosomal damage by inducing the formation of micronuclei in bone marrow cells, and significantly (*p* < 0.05) higher levels of micronucleated polychromatic erythrocytes were observed compared to the control groups. In conclusion, 2,5-HD causes oxidative and chromosomal damage characterised by micronuclei formation and DNA fragmentation [85].

In assessing the carcinogenic effects of *n*-hexane, the U.S. agency EPA concluded in 2005 that the available database did not contain sufficient information to evaluate the carcinogenic potential. In the main animal study, an increased incidence of combined adenomas and hepatocellular carcinomas was observed in female rats exposed to commercial hexane (mixture of isomers), with a statistically significant trend towards an increased incidence of pituitary adenomas. However, these effects were not observed in male mice or in either sex of F344 rats exposed to commercial hexane under the same conditions [47]. In addition, only one case-control study was available that examined the association between the occurrence of brain tumours in workers at a petrochemical plant and exposure to chemical and physical agents, including *n*-hexane. The work by Beall et al. (2001) suggested that occupational exposure may have contributed to the excess incidence of gliomas among workers, but no association was found between exposure to *n*-hexane and the incidence of brain tumours. These results, taken together, do not allow definitive conclusions to be drawn on the carcinogenicity of this solvent [97]. As far as these authors are aware, there are no other available studies investigating the possible carcinogenic effects of *n*-hexane.

As mentioned above, the toxicity of *n*-hexane is not limited to the nervous system but extends to multiple organs. Potential toxic effects on the immune system were highlighted in the study by Karakaya et al. (1996), which examined the relationship between exposure to *n*-hexane and various markers of immune function in 35 occupationally exposed male workers. Significant suppression of serum immunoglobulin levels (IgG, IgM, and IgA) was observed in the exposed group compared to the control group, with a significant correlation between the 2,5-HD urine concentrations and serum immunoglobulin levels in the exposed group. In addition, no significant difference was found between the white blood cell counts in the two groups [86].

Pulmonary toxicity was confirmed by Bouakkaz et al. (2018) after oral exposure of rats to *n*-hexane. In the exposed groups, lung weight increased significantly, and histological findings revealed pneumonia characterised by bronchopneumonia, alveolar lesions, congestion, haemorrhage, pneumocyte hyperplasia, bronchial epithelium degradation and inflammation. Pulmonary toxicity was correlated with significantly increased levels of white blood cells, lymphocytes, granulocytes and eosinophils [87].

In addition to pronounced neurotoxicity, 2,5-HD has been associated with alterations in the blood-nerve barrier (BNB), the physiological boundary between peripheral nerve axons and the bloodstream that regulates the transfer of substances from the plasma to the endoneurial compartment. In the study by Liu et al. (2010), the permeability of the BNB was increased in rats exposed to 2,5-HD (400 mg/kg once daily via gavage for four weeks). In animal models of neuropathy, there is evidence that changes in the BNB may precede and follow the degeneration of nerve fibres or demyelination. However, the mechanism underlying the increase in BNB permeability is still not clear, and it is still unclear whether this process precedes the histological or functional evidence of 2,5-HD-induced neuropathy [88].

In a study on rats, Adedara et al. (2014) analysed the mechanisms of 2,5-HD toxicity in the liver and kidney. After 21 days of exposure to various concentrations of orally administered 2,5-HD (0.25, 0.5, and 1% in drinking water), histological examinations showed central venous congestion and cellular infiltration by neutrophils in the liver and progressive degeneration of the proximal renal tubules characterised by mild haemorrhages in the interstitium of the tubular epithelial cells with severe vacuolisation and renal tubular necrosis. Increases in serum ALT, AST and alkaline phosphatase (ALP) observed in rats exposed to 2,5-HD confirmed liver damage and alteration of the hepatobiliary system. In addition, increased levels of total and conjugated bilirubin indicated a possible disruption of bile flow in the biliary system. The reduced renal function was evidenced by a significant increase in plasma urea and creatinine levels in rats exposed to 2,5-HD, with a significant increase in electrolyte levels indicating a disruptive effect of 2,5-HD on ion-dependent processes in the kidney. The increased activity of several antioxidant enzymes (SOD, GPx, GST), which was accompanied by a marked increase in H_2_O_2_ and MDA concentrations in the liver and kidney, underlined the key role of oxidative stress in the toxicity induced by 2,5-HD [89].

### 4.2. Epidemiological Studies on Non-Occupationally Exposed Population

*n*-Hexane is considered a ubiquitous pollutant due to its worldwide use as a solvent in various industrial processes, being one of the most common components in industrial products containing solvent residues, including the food industry. Moreover, it is present in gasoline at a concentration of about 3%, and some studies confirmed its constant presence in the air, as described by Mandin et al. (2017), who assessed air quality in 140 office buildings in Europe and found indoor concentrations of *n*-hexane as high as 9.1 μg/m^3^ in summer and 8.6 μg/m^3^ in winter [98]. As already stated, among the various indicators, the 2,5-HD urine levels are a sensitive and reliable biological indicator of *n*-hexane exposure [99]. Acid hydrolysis of urine, which is performed to quantify total 2,5-HD, also converts non-toxic metabolites to 2,5-HD, and the yield varies with pH, whereas to determine free 2,5-HD, the sample is not hydrolysed. Since the correlation between *n*-hexane exposure levels and total 2,5-HD concentration is stronger [56], this is probably a better indicator for assessing the extent of contact, while free 2,5-HD is a more accurate indicator for assessing exposure risk since its concentration is directly related to neurotoxic effects [100]. The free 2,5-HD content in urine was adopted for the Biological Exposure Indices (BEI) to control occupational health risks. The American Conference of Governmental Industrial Hygienists (ACGIH) reported a value of 0.5 mg/L at the end of the shift [101]. However, a level of 2,5-HD in urine is not only detected in workers occupationally exposed to *n*-hexane but is also easily found in the general population, as described in Table 3 [25,57,66,70,100,102,103,104,105,106,107]. Several factors have been shown to be correlated with 2,5-HD urine levels. Cigarettes contain large amounts of aromatic and aliphatic hydrocarbons, including *n*-hexane, and higher 2,5-HD levels have been found in the urine and blood of smokers than in non-smokers [70,100]. However, Salamon et al. (2019) observed higher concentrations of free 2,5-HD in smokers [102] but without finding a statistically significant difference from controls, while Persson et al. (2013) found no differences [66]. The results showed that urinary excretion of total 2,5-HD was also significantly higher in men than in women [66,100,103], while no sex difference was found for urinary excretion of free 2,5-HD, probably because metabolic differences are only observed for the metabolite resulting from acid hydrolysis [102]. In addition, urinary excretion of 2,5-HD correlated with age, with lower excretion of 2,5-HD in the elderly [66,100]. Furthermore, in the study by Salamon et al. (2019), urinary levels of free 2,5-HD were significantly higher in the urine of individuals exposed to low-intensity vehicle traffic than in individuals exposed to moderate-intensity. Inhibition of the biotransformation of *n*-hexane by concurrent exposure to toluene or xylene from urban traffic has been reported in the literature, which may explain this observation. However, the intensity of vehicle traffic was not determined by objective measurements but only by means of a questionnaire. Further research would be needed to understand if there is a causality. 2,5-HD levels were higher in the urine of overweight individuals than in normal-weight persons, and this result may be related to the high distribution of *n*-hexane in adipose tissue, which delays its excretion and leads to its accumulation. Anyway, the authors recommend that these results need to be confirmed in a larger population. Moreover, the questionnaire proposed in this study did not address dietary habits, which could also contribute to exposure to hexane, given its massive use in the food industry and the possible presence of its traces in the final products.

In the studies reported, free 2,5-HD averaged between 8% and 18% of the total 2,5-HD detected in urine. Given the presence of 2,5-HD in the urine of subjects not occupationally exposed to *n*-hexane or MBK, it is important to understand the possible cause of this detection. Perbellini et al. (1993) estimated the daily pulmonary intake of *n*-hexane to be 10–100 µg/24 h based on the values reported in the literature for indoor *n*-hexane of 5–50 µg/m^3^ and concluded that these values could not explain the high concentration of 2,5-HD in the urine of the general population. The authors hypothesised a potential endogenous source of aliphatic hydrocarbons generated by lipid peroxidation and suggested that only a small fraction of the detected 2,5-HD might originate from *n*-hexane as a ubiquitous micropollutant [105]. However, as suggested by Persson et al. (2013), environmental microexposure cannot be ruled out [66], which may not be related only to inhalation exposure to *n*-hexane in the air. Given the widespread use of this solvent at the industrial level, chronic exposure to low concentrations could potentially occur via various industrial products, including some foods that have been shown to contain traces of *n*-hexane, as described in the following section.

### 4.3. n-Hexane Residues in Commercial Products

The crushing industry uses “food grade” hexane, which contains a low percentage of benzene that cannot be completely removed and, therefore, must be kept below a certain limit (e.g., 1000 mg/kg solvent for ASTM); see Table 1 [28]. Toluene is another toxic compound found as an impurity in hexane, but unlike benzene, no concentration limit has been set for food-grade hexane. The designation “food grade” is not linked to health safety standards but simply refers to a manufacturing process that includes an additional purification step to decrease the level of aromatic compounds which are naturally high in naphtha.

According to the Best Available Techniques Reference Document for the Food Industry (BAT), despite efficient solvent recovery, hexane losses occur in several steps of the extraction cycle in oilseed processing [30], as shown in Table 4.

Hexane recovered in meals is the main cause of losses, accounting for 30–60% of total losses, and this percentage depends mainly on the physical properties of the matrix. For crude oil, the hexane loss is 2–6% of the total losses, which shows that a significant amount of hexane is recovered in these two products. Finally, feed meals are directly sold to feed manufacturers, while oil and protein are refined for human consumption and then supplied to food manufacturers.

Some studies have been conducted on *n*-hexane residues in commercial products showing the presence of *n*-hexane in commercial hexane-extracted oils, in food products, and in functional health foods, as shown in Table 5.

Yousefi et al. (2017) investigated *n*-hexane residues in 40 edible oils collected from the Iranian market and found them in 90% of the samples (36 out of 40), with hexane levels above the limit of quantification (LOQ) in 14 samples. This study showed that most oils contained hexane residues; however, the level in all samples was below the European MRL of 1 mg/kg, namely 0.043 mg/kg, which was found in a commercial rapeseed oil [108]. Olive pomace oil, called Orujo oil in Spain, is obtained by extraction with hexane from the olive residues left after pressing the fruit to produce virgin olive oils. Using a headspace sampler directly coupled to a mass detector, Peña et al. (2003) detected *n*-hexane residues in two oils at concentrations of 2 and 3 mg/kg, respectively. Although these values were above the current European MRL, hexane was not detected in most samples, but this was probably due to the high detection limit (LOD) of the instrument (0.7 mg/kg) [109]. Michulec and Wardencki (2004) investigated the presence of hexane, benzene, and toluene in different primrose oils with an HS-GC-FID and found traces of *n*-hexane in nine samples with a maximum concentration of 0.70 mg/kg. Furthermore, the benzene content in all samples was below the LOD, while traces of toluene were detected in nine samples, with levels above the LOQ in three samples reaching a maximum value of 0.18 mg/kg [110]. In two other products, cardamol and synthetic vitamin A, the residual hexane content was quantified, with values of 0.78 and 6.10 mg/kg, respectively. Of the 87 oils analysed by Oh et al. (2005), nine showed traces of *n*-hexane and eight of them at a concentration higher than LOQ (0.5 mg/kg). Using an HS-GC-FID with an automatic sampler, levels higher than 1 mg/kg were detected in two oils (soybean oil and Chinese pepper oil), and in margarine samples. Moreover, using a manual-injection method, hexane levels up to 11.2 mg/kg were found in margarine samples, but these high values are possibly due to a poor sampling homogeneity of the solid product, confirmed by a significant difference between the values obtained by manual-injection and auto-sampling [111]. Ligor and Buszewski (2008) focused on the determination of residues of VOCs in 16 vegetable oils by comparing two different sample preparation techniques, SPME and static headspace (SHS). *n*-Hexane residues were found in almost all samples, with the exception of oils obtained by mechanical pressing. Both sample preparation techniques yielded very similar results, and hexane concentrations ranging from 0.005 to 0.400 mg/kg were recorded by SPME-GC-FID. In addition, benzene residues were found in three samples (in one of them at a concentration close to LOD) with a maximum level of 0.607 mg/kg. The concentration of toluene, which is contained in food-grade hexane, was above the LOQ in five samples, with a maximum value of 0.065 mg/kg [112]. These results clearly show that during oil extraction with hexane, along with this hydrocarbon, other toxic and carcinogenic substances could directly enter the food chain and end up in the final consumer products. In 2018, Ramezani et al. analysed ten different oils collected on the Iranian market. Among these, six products contained traces of hexane and all of them at concentrations above the European MRL up to 11.10 mg/kg, while one oil also contained benzene residues [113]. The residual hexane content in commercial products of the four main types of oil produced in Malaysia was assessed by Samsuri et al. (2021) [114]. All the analysed commercial oils contained solvent residues, with the highest content found in a sunflower oil (2.688 mg/kg of hexane). Hexane was detected in several commercial edible oils, in some cases at concentrations above the European MRL of 1 mg/kg. As far as the authors are aware, the hexane content in food products manufactured in Europe is not monitored by any authority, nor is it monitored in imported products, which may lead to dangerous exposure of citizens to this toxic solvent.

In the oilseed crushing industry, the other products that can potentially lead to hexane or 2,5-hexanedione entering the food chain are defatted meals used as animal feed. According to the feed additive regulation EC 1831/2003 [117], hexane is a “processing aid” and may result in the “unintentional but technologically unavoidable presence of residues” in the final product, provided that these residues do not have an adverse effect on human and animal health and the environment. The limit of hexane in feedstuffs (1000 mg/kg) is considerably higher than in edible oils, and there are few studies on the possible presence of hexane in products derived from animals fed with hexane-defatted meals. As the toxicity of hexane is mainly caused by long-term exposure and an official reference dose for chronic oral exposure (RfD) has not yet been established, it is difficult to scientifically demonstrate that such residues are safe for human health.

Over the past 20 years, people’s growing interest in wellness through nutrition, an ageing population and skyrocketing healthcare costs have dramatically boosted sales of nutraceuticals and health food products. The global nutraceutical market, fuelled by the sale of nutraceutical beverages, foods, and supplements, has grown at a rate of 7.3% from 2015 to 2021 and will be worth approximately $280 billion by the end of 2021 [118]. Among the wide variety of nutraceuticals available on the market, vegetable oils and fats play a key role as they are a good source of essential fatty acids and other minor components, such as lecithin, tocopherols and tocotrienols, carotenoids, phytosterols, etc., that have various beneficial effects on human health [119]. Many vegetable oil-based nutraceuticals are derived from oils extracted with hexane, and hexane is also used to extract or separate functional raw materials in the production of health functional foods. Consequently, even these compounds, which are believed to have only beneficial effects, may contain traces of hexane, as documented by Jeong et al. (2017). In this study, hexane residues were determined in 60 health food samples from ten different product classes. Traces of hexane were detected in nine of the ten product classes, with higher mean values in products containing phosphatidylserine, conjugated linoleic acid, and lecithin [115].

As mentioned above, hexane is still commonly used as a solvent to obtain natural extracts. The European and U.S. regulations are ambiguous regarding the use of the term “natural”, without mentioning any ban on the use of hexane in the production of natural products. Ito et al. (2012) adopted an HS-GC-FID analytical method for the quantification of residual solvents in annatto extracts, used as natural food colourants, and found hexane residues in 2 of the 23 analysed samples [116].

Current analytical methods for the quantification of hexane residues have a high LOQ. For example, at the French Reference Institute for Oils and Fats (ITERG), the LOQ for hexane is 1 mg/kg in liquids (oils). With a LOQ of 1 mg/kg in oils, it is not possible to accurately measure the number of hexane residues in oils if they are present in trace amounts. Residual amounts of hexane are easily found in many hexane-extracted oils, and since vegetable oils have a wide variety of food applications, cumulative exposure to hexane residues may occur. Even though oils with a hexane concentration of less than 1 mg/kg are legally compliant, the few studies on the effects of chronic oral hexane intake do not provide solid scientific evidence that ingestion of low levels of hexane over a prolonged period is safe for human health.

## 5. Solutions Exist to Replace Hexane as Extraction Solvent

In recent decades, various efforts have been made to replace toxic VOCs in chemical processes, according to the principles of Green Chemistry and Green Extraction. To this end, various approaches have been explored involving both the use of non-toxic solvent systems and green extraction intensification technologies, as described in the following sections.

### 5.1. Solvent-Free Extraction

Do we really need a solvent? Before we start looking for safer solvents, we should always ask ourselves this question because “the most environmentally friendly solvent in terms of waste reduction is no solvent”, as Kerton and Mariotte (2013) pointed out [120]. One of the most well-known solvent-free processes from ancient times to the present day is the extraction of olive oil by mechanical pressing. Olive oils are extracted from the fruit of the olive tree using purely physical methods, including crushing the olive fruit and mixing and separating the olive oil from the resulting paste. This process has many advantages, including the simultaneous extraction of lipophilic and hydrophilic compounds, such as lipids with natural antioxidants that inhibit lipid autoxidation, but also many volatile and non-volatile compounds responsible for aroma and flavour.

**Figure 5:**
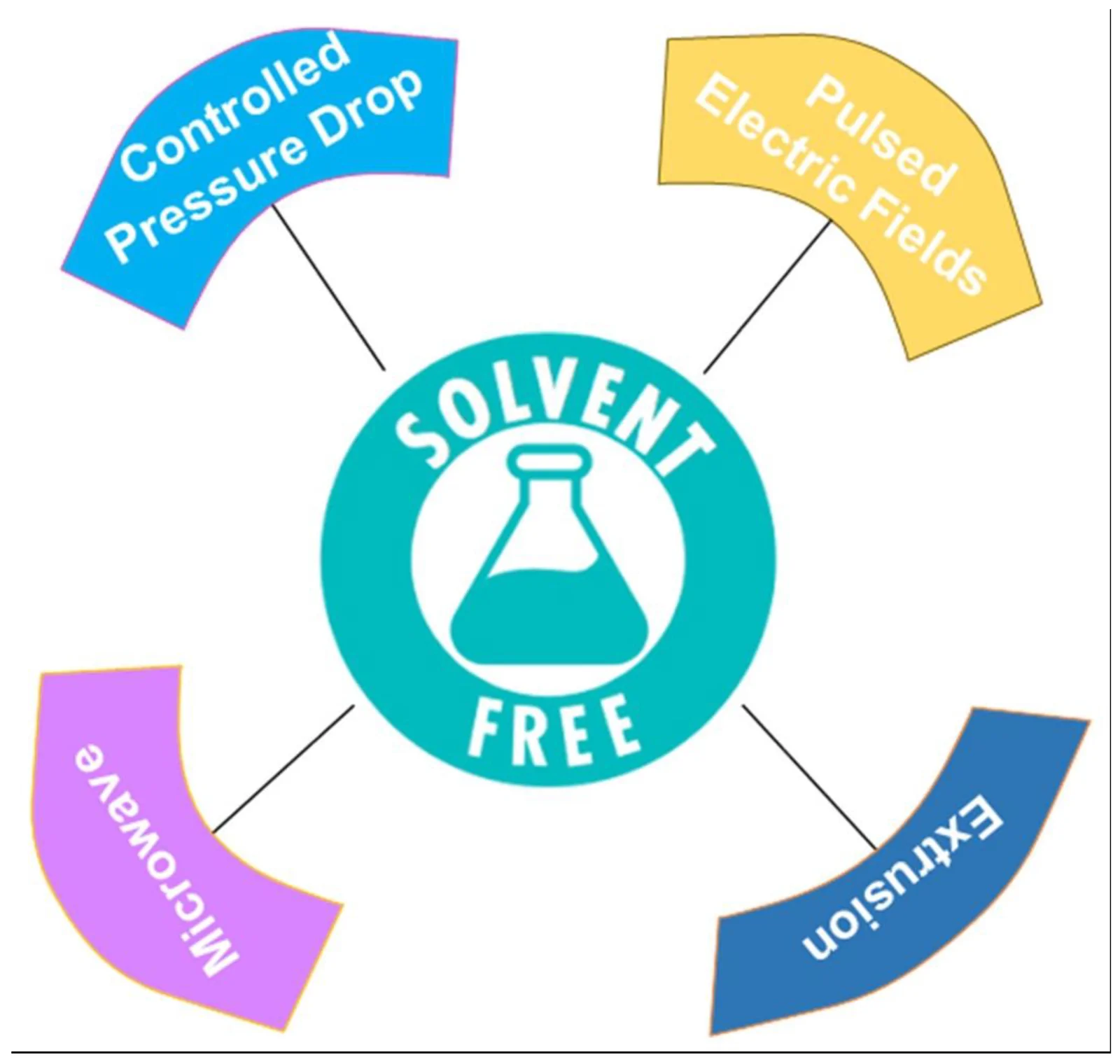
Different solvent-free extraction technologies.

In the 18th century, cold pressing was also used as an extraction method for essential oils, or more specifically for essences, contained in citrus peels. In this technique, the pericarp or “zests” (also called flavedo) is pressed so that the essence contained in the oil pockets lining the peel of the fruit flows outwards and can be collected. In recent years, the development of solvent-free processes has gained increasing interest to modernise conventional petroleum-based organic solvent extraction processes. Various technologies such as Instantaneous Controlled Pressure Drop (DIC), Pulsed Electric Fields (PEF) and Microwave Irradiation (MW), as well as extrusion (using hydraulic presses or screw presses), have been successfully used for solvent-free extraction of primary and secondary metabolites (essential oils, flavours, edible oils, antioxidants and other organic compounds) (Figure 5) [121]. These innovative techniques enable practical and efficient extraction and reduce the extraction time from several hours to a few minutes by avoiding the distillation of the solvent, the limiting step of the process. In addition, no wastewater is produced after treatment and energy consumption is usually lower than with conventional solvent extraction. The positive features of solvent-free extraction are numerous: (i) reducing the costs and risks associated with the use of organic solvents; (ii) facilitating scale-up; (iii) improving safety by reducing overpressure and explosion risks [122].

### 5.2. Water as a Green Solvent

**Figure 6:**
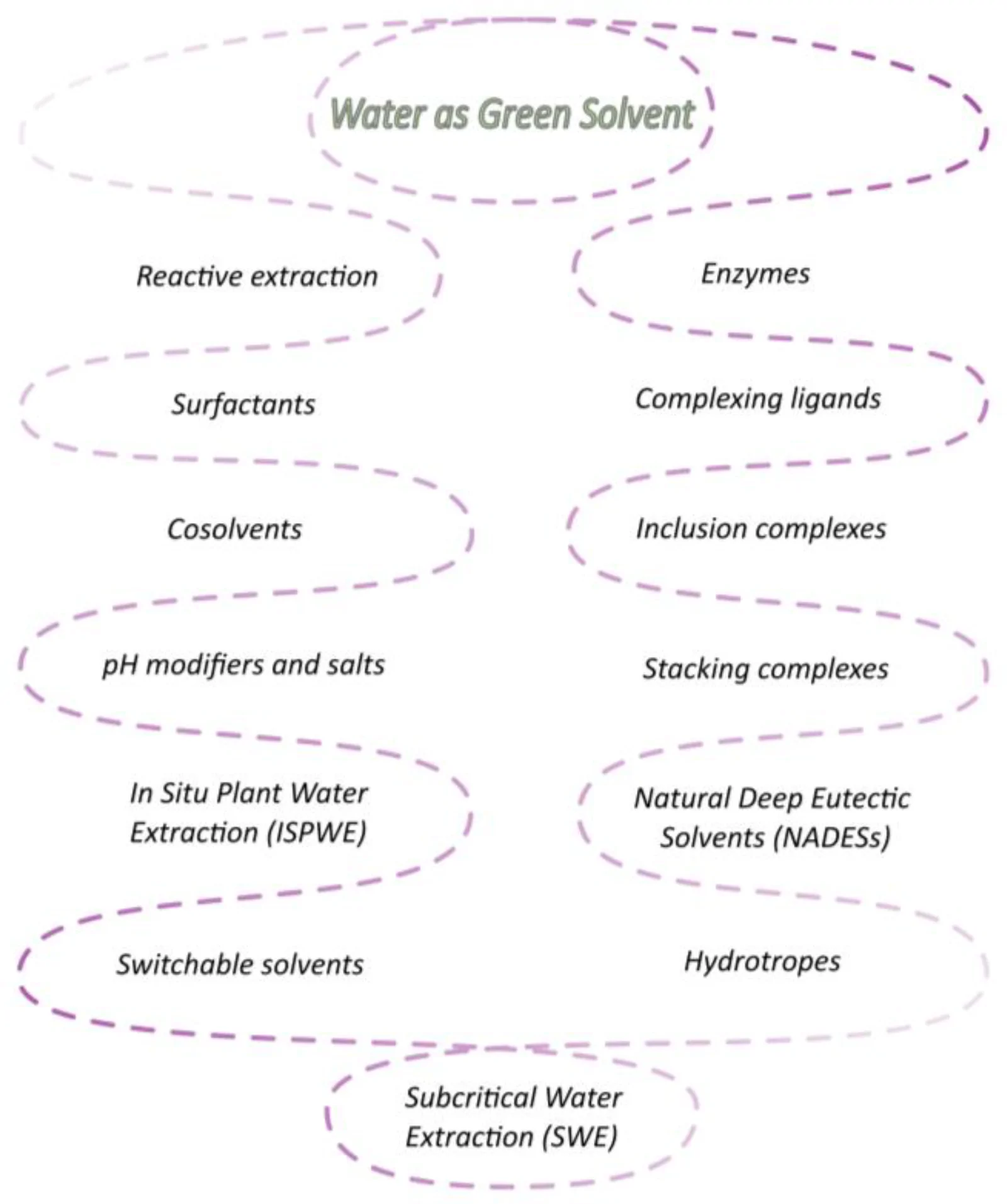
Water as green solvent.

From a “natural” point of view, water is the most environmentally friendly solvent. Not only is it cheap and environmentally benign, but it is also non-toxic and non-flammable, providing opportunities for clean processing and pollution prevention. However, when water is used for solubilisation or extraction of natural products (NPs) from real biological matrices, this solvent appears to be relatively inefficient. In fact, it is almost impossible to solubilise or extract many natural products using only water, as they are poorly soluble in this solvent. To compensate for this low efficiency in solubilising or extracting NPs, researchers have developed various methods to improve the solvent potential of water while taking advantage of its environmentally friendly properties. Figure 6 summarises some of the most important methods for improving the solvent potential of water with the aim of more efficient solubilisation and extraction of natural products. Some of these methods are based on the addition of a chemical agent (organic, inorganic, biochemical), including pH modifiers and salts, co-solvents, surfactants, complexing ligands, inclusion complexes, stacking complexes, hydrotropes, natural deep eutectic solvents (NADESs) and enzymes. Other methods based on the addition of a chemical agent are reactive extraction and switchable solvents. In addition, some technologies involve appropriate physical treatment of the water, namely in situ plant water extraction (ISPWE) and subcritical water extraction (SWE), which exploits the change in the dielectric constant of water under subcritical conditions.

In water extraction of plant matrices, the application of extraction technologies such as microwaves, ultrasound, hydrodynamic cavitation, pressurised reactors for subcritical water extraction, PEF and enzymatic treatments have paved the way for the expansion of studies and the development of new pilot and semi-industrial reactors. During plant matrices extraction, a partially damaged cell wall greatly improves solvent accessibility for both intracellular solutes and cell wall components. Hydrolytic enzymes are used to selectively depolymerise and degrade certain cell wall components such as pectin, cellulose and hemicellulose. In aqueous-based extraction processes, incubation with enzymes can take place simultaneously with the mixing step. Enzyme treatment leads to a reduction in solvent consumption and extraction time, while mild operating conditions favour the good recovery of thermolabile products [123]. Cavitation is the formation, growth and disintegration of gaseous bubbles in a liquid. This process can be triggered by acoustic (ultrasound) or mechanical (hydrodynamic) waves that create cycles of compression and expansion. During these cycles, the bubbles can reach their critical size and then collapse, releasing a large amount of energy and creating a microenvironment that reaches 5000 K and 1000 bar. Cavitation technologies using ultrasound, hydrodynamic rotor/stator, or high-shear homogenisers significantly increase extraction yields in aqueous solvents [124].

PEFs are high voltage (10–50 kV/cm) and very short (<10 μs) electrical pulses generated by high voltage high current switches. PEF can be used as a pre-treatment or directly for extraction as it can cause electroporation, which opens pores in cell membranes, and cell rupture, both mechanisms that greatly enhance the recovery of intracellular compounds [125]. Microwaves are mainly used for selective and rapid heating and offer lower energy consumption than conventional technologies. One efficient technique is microwave-assisted hydrodistillation, which is used for the extraction of essential oils, and microwave gravity hydro diffusion, which has shown promise as an extraction technique for the simultaneous recovery of essential oils and other compounds such as pigments and polyphenols [126]. Although the dielectric properties of water mean that only polar products can be extracted from plant matrices, limiting their use, water can be used in a subcritical state to overcome these limitations. In the temperature range between 100 °C and its critical temperature (374 °C), the properties of water can be changed, provided it is kept in the liquid state by sufficient pressure. As the temperature increases, the dielectric constant of water decreases, reaching values similar to those of ethanol at 250 °C, which allows the extraction of less polar compounds. The viscosity also decreases, allowing better penetration into matrices, while the ion product increases, favouring the depolymerisation of complex structures [127]. SWE has been shown to be a suitable method for the additional extraction of lipophilic compounds, although the method requires further purification due to low extraction selectivity. Indeed, due to the high extraction efficiency of SWE, the primary (proteins, fats, carbohydrates and soluble fibres) and secondary metabolites are extracted in a very efficient way.

### 5.3. Green Solvents: From Ionic Liquids (ILs) and Deep Eutectic Solvents (DESs) to Natural Deep Eutectic Solvents (NADESs)

The search for alternatives to VOCs has led to great interest and growing demand for ionic liquids (ILs). ILs are generally defined as a group of non-molecular solvents produced by the combination of organic cations and organic or inorganic anions that melt below 100 °C [128]. Many ILs have some interesting properties, such as non-flammability, thermal stability, low vapour pressure and, above all, impressive synthetic tunability and versatility [129]. These solvents have long been recognised as designed green solvents. However, in recent years, their “green” aspect has been largely questioned due to their poor biocompatibility and biodegradability [130,131]. Deep eutectic solvents (DESs) have been slowly developed since 2004 as a greener alternative to ILs to circumvent this problem [132]. DESs, commonly defined as a subclass of ILs, can be prepared by mixing solid compounds to form a eutectic mixture with a melting point lower than the melting point of each individual component [133].

**Figure 7:**
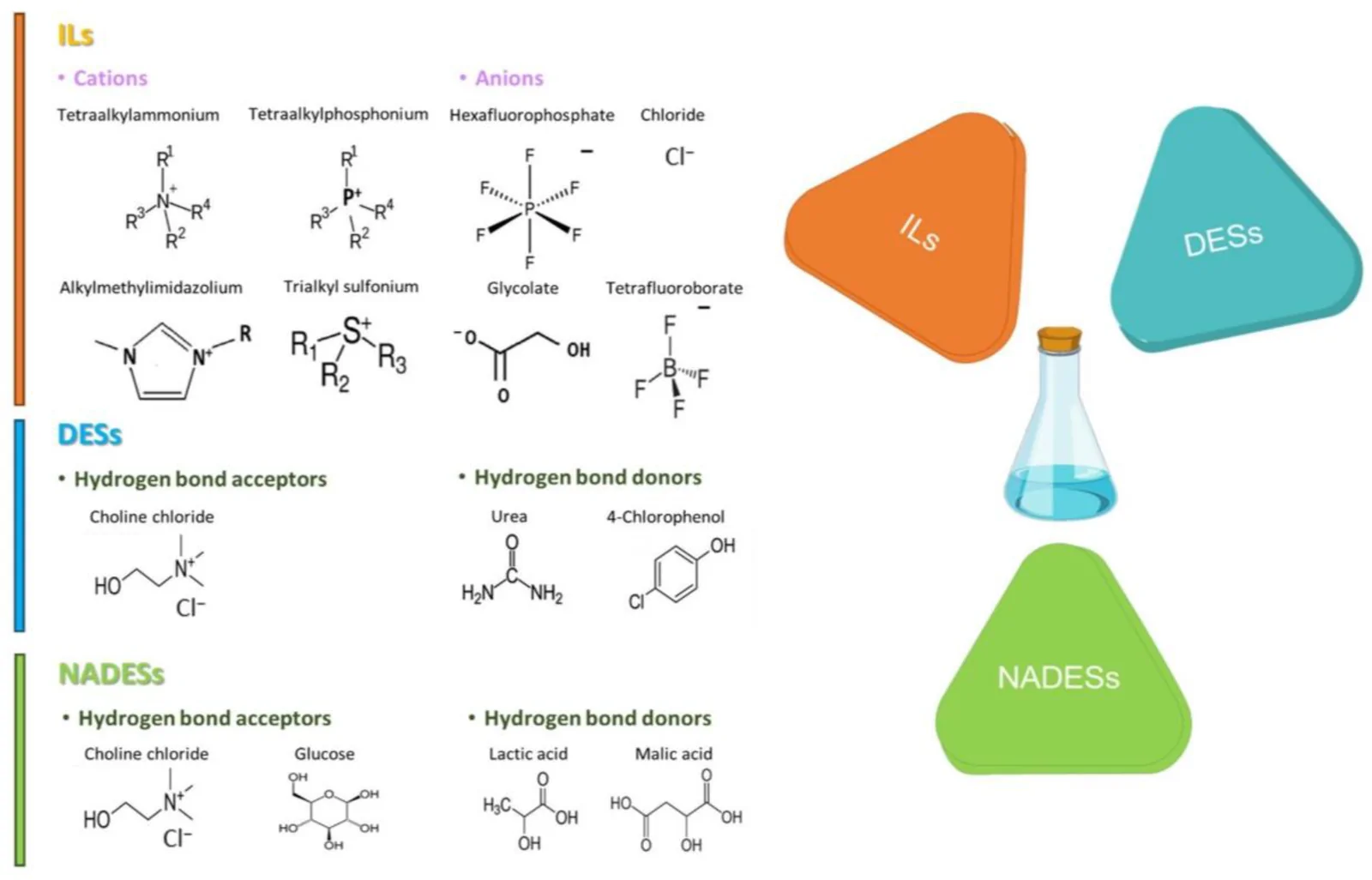
Most common components of ILs, DESs and NADESs [122].

In response to the principles of Green Chemistry proposed by Anastas and Warner (1998) [134], natural sources of DESs have attracted much attention as substitutes for synthetic compounds [135], giving rise to a new class of DESs, namely Natural Deep Eutectic Solvents (NADESs). Like DESs, NADESs are mixtures of compounds that have a much lower melting point than their individual components [136]. In addition to all the advantages of DESs, NADESs are considered environmentally friendly and “readily biodegradable” due to the natural origin of their components [132,136], so that the extracts obtained can be safely used in the food, pharmaceutical and cosmetic industries [137]. The known compounds that form this liquid phase are primary metabolites such as organic acids (lactic, malic, citric acid, etc.), sugars (glucose, fructose, sucrose, etc.), amino acids, choline chloride, etc. [131,138]. The main difference between ILs and DESs (including NADESs) is the intermolecular strength, which is based on ionic bonds in ILs and hydrogen bonds in DESs and NADESs. Compared to ILs, DESs and NADESs showed much lower toxicity. Thanks to this feature NADESs have been approved as component of several cosmetic formulations (Figure 7).

### 5.4. Bio-Based Solvents

**Figure 8:**
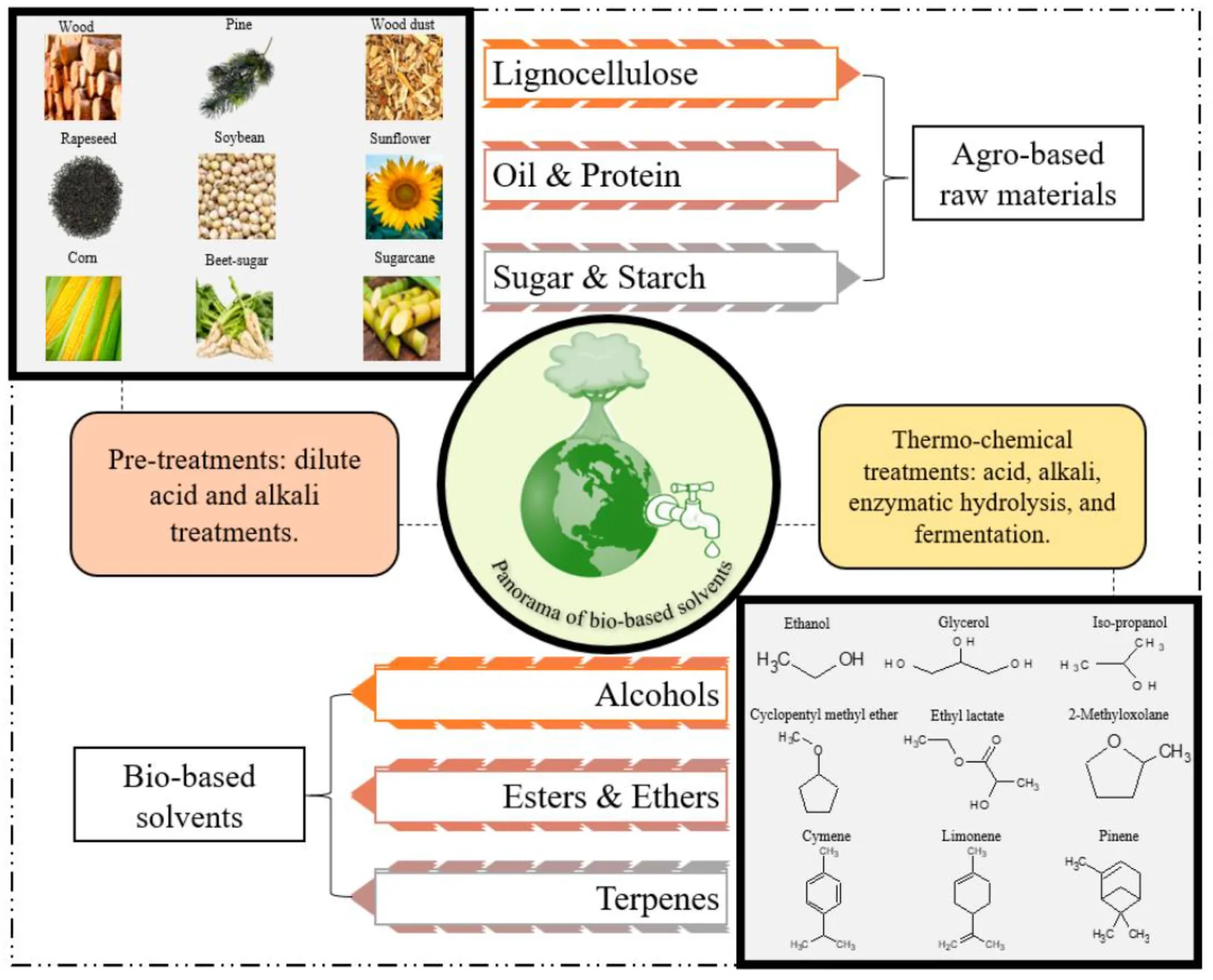
Bio-based solvents scenario [122].

Bio-based solvents, as the name suggests, are produced from biomass, usually from carbohydrate-rich agricultural crops such as corn, wheat or beet, or from organic residues that have long been considered waste, although these are not the only sources. Depending on the origin of the biomass used to produce these solvents, they can be divided into four categories: (a) lignocellulose; (b) sugar and starch; (c) protein and oil; and (d) other forestry and food wastes. Solvents derived from these categories can be further classified according to their functional groups: esters (e.g., ethyl lactate and levulinate), ethers (e.g., ɣ-valerolactone and 2-methyloxolane), terpenes (e.g., limonene and cymene) and alcohols (e.g., bioethanol), or by the petroleum-based solvent they are intended to replace (Figure 8).

To be identified as a green solvent, this should not only ideally fulfil the twelve criteria proposed in the principles of Green Chemistry, but also meet certain prerequisites. To name a few: the solvent should be derived from renewable raw materials, be recyclable through eco-efficient processing, show solvent properties similar to those of conventional solvents it is expected to replace (e.g., boiling point and vapour pressure), as well as increased biodegradability under normal environmental conditions [139,140]. In addition, the production or synthesis of bio-based or green solvents should not have negative environmental and health impacts. Agricultural biomass used for solvent production has a rigid and fibrous structure because it is treated after harvesting. Three processes are usually used to produce solvents from biomass: fermentation, chemical conversion of biomass derivatives, and use of waste materials from other processes. Powerful thermochemical and/or catalytic processes supported by pre-treatments, therefore, allow efficient structural changes of biomass to produce suitable solvents.

### 5.5. Supercritical Fluids and Liquefied Gases

**Figure 9:**
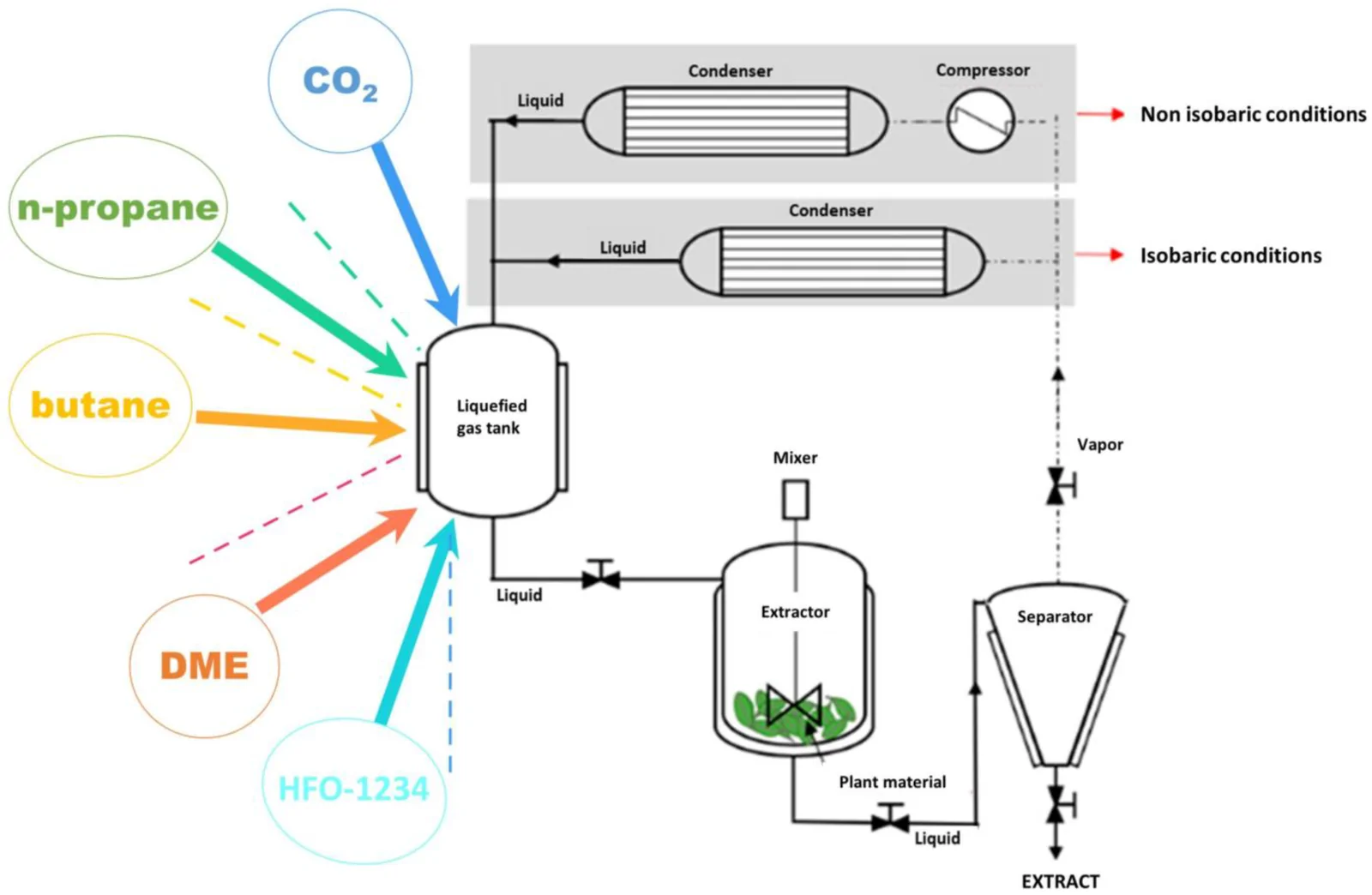
Process diagram of a unit designed for extraction using CO_2_ or liquefied gases.

Supercritical fluids (SCFs) are a well-established alternative to conventional organic solvents [141]. A fluid reaches its critical state when two phenomena occur simultaneously: (1) it is heated above its critical temperature (Tc), and (2) it is pressurised above its critical pressure (Pc). The physical properties of SCFs (viscosity, density and diffusivity) can be easily tuned by adjusting temperature and pressure conditions. The pressures used in industrial plants (e.g., in supercritical CO_2_ extractors) are often considerably higher than the solvent-critical pressure. At higher pressures, an increase in SCFs density is achieved without a significant increase in viscosity. The liquid-like density of SCFs leads to a solvation force close to that of liquids, while their gas-like viscosity leads to high mass transfer. Carbon dioxide (CO_2_) is the most widely used SCF, as it is inert, non-toxic, non-flammable, inexpensive, abundant, easily removed from the product and has moderate critical properties (Tc = 31.1 °C, Pc = 7.38 MPa). The use of supercritical CO_2_ extraction techniques allows efficient extraction of lipophilic compounds at generally mild temperatures (30–70 °C) without leaving traces in the extract. Since supercritical CO_2_ is a non-polar solvent, its solvation capacity is known to be between that of pentane and toluene [142]. Usually, a polar co-solvent such as methanol or ethanol can be added to improve the solubility of polar substances. CO_2_ is a solvent generally recognised as safe (GRAS), so products containing extracts obtained with “food grade” CO_2_ are safe for human health. Numerous studies have been conducted on the extraction of natural products with supercritical CO_2_ [143]; however, the high pressures required to reach the supercritical state limit its use to very high value-added products, as the investment costs for an industrial plant are correspondingly high. This obstacle to the development of large-scale supercritical CO_2_ processes has led to liquefied gases becoming valuable alternatives to conventional solvents, as they also allow the extraction of lipophilic compounds at room temperature without leaving traces in the final product. In contrast to SCFs, the use of liquefied gases requires moderate pressures (1–10 bar), which results in lower energy consumption and facilitates their application on an industrial scale. Depending on the pressure and temperature conditions, the dissolving power can be adjusted by changing the density, which enables selective extractions. The use of liquefied gases in a process that can be easily transferred to the industrial scale requires certain selection criteria related to technical considerations. In particular, the following two thermodynamic criteria are crucial for their suitability for industrial use: (i) a boiling temperature at atmospheric pressure between −30 °C and +20 °C so that the gas can be condensed at temperatures that are reasonably achievable on an industrial scale; (ii) a vapour pressure of less than 10 bar at room temperature, in order to limit the investment costs for pressure equipment. The most commonly used liquefied gases are propane, *n*-butane, dimethyl ether (DME), 1,3,3,3-tetrafluoropropene and 2,3,3,3-terafluoropropene (HFO-1234yf) (Figure 9). The main advantages of liquefied gases over other conventional solvents, such as SCFs and liquid solvents (water and organic solvents), are as follows: (i) they can be used at low temperatures; thanks to their low boiling point, liquefied gases can be evaporated at moderate temperatures, without risk of degradation of the molecules of interest (including volatile ones), and without leaving traces of residual solvents; (ii) they can be used at moderate pressure; compared to SCFs, liquefied gases are generally used at much lower pressures, between 1 and 10 bar, which limits investment costs; (iii) under “normal” solid-liquid extraction conditions, most liquefied gases are chemically inert. Depending on their chemical structure, liquefied gases have particular solvation properties that condition their choice as solvents for the extraction of hydrophilic or lipophilic molecules.

## 6. Conclusions and Future Trends

Hexane is a rather confusing name for a mixture of molecules whose composition can vary depending on the petroleum used as feedstock and the refinery’s processing strategy, but which consists mainly of *n*-hexane and contains impurities such as benzene. The demand for this solvent is increasing year by year as it has a wide range of applications. The predominant industrial use is in the food industry and in particular for the production of vegetable oils, flavours and fragrances, colour additives or other bioactive ingredients. As a result, hexane can enter the food chain through a variety of edible end products. Although the human toxicity of hexane was reported several decades ago, there are only a few studies that allow a preliminary determination of an oral reference dose (RfD) and inhalation reference concentration (RfC). Furthermore, these studies are associated with intermediate or low confidence levels, which means that the real toxicity of this substance may be underestimated. It is well documented that the toxicity of *n*-hexane is mainly due to its major metabolite 2,5-hexanedione (2,5-HD). The toxicity of *n*-hexane to the nervous and reproductive systems has been demonstrated in several studies. In addition, recent studies have suggested new toxic effects of this solvent, including a possible link between exposure to *n*-hexane and Parkinson’s disease or endocrine disruption.

Moreover, a level of 2,5-HD in urine is not only detected in workers occupationally exposed to *n*-hexane but is also readily found in the general population. As environmental microexposure to *n*-hexane cannot be ruled out, exposure through ingestion of residues in food should also be considered. Residual amounts of *n*-hexane have been reported in various foods, especially vegetable oils. Since vegetable oils have a wide variety of food applications, cumulative exposure to *n*-hexane residues may occur. Although oils with a hexane concentration of less than 1 mg/kg are legally compliant in the EU, the few studies on the effects of chronic oral hexane intake do not provide solid scientific evidence that the intake of low levels of hexane over a prolonged period of time is safe for human health. Therefore, we hope that this review will support an updated re-evaluation of the use of hexane in food by the authorities. There are several alternatives to hexane for the extraction of natural products, including solvent-free extraction, water, NADESs, bio-based solvents, supercritical fluids or liquefied gases. However, the increasing demand for hexane from year to year suggests that there is no real incentive in industry to replace this toxic solvent. As hexane is currently considered a “processing aid”, it is exempt from labelling on food packaging, and consumer awareness of its still extensive use in the food industry is likely to be extremely low. This lack of public concern may be the main reason why this substance is still in use more than 50 years after it was shown to be neurotoxic to humans. Better labelling with an obligation to indicate on food packaging the use of hexane as a “processing aid” could provide the incentive the industry needs for substitution.

In this context, we hope that this review will help to improve academic and public knowledge and stimulate further scientific work to identify, assess and prevent the adverse effects of hexane on human health. We believe that substituting the use of hexane in the food industry could lead to a significant reduction in population exposure and has the potential to improve the health status of our fellow citizens worldwide.

## Figures and Tables

**Table 3:** Concentrations of 2,5-HD in urine of non-occupationally exposed population.

Country	n	Mean Total 2,5-HD mg/L (Range)	Mean Free 2,5-HD mg/L (Range)	References
Mexico	32	N.A.	0.043	[25]
Italy	99	0.18 (0.06–0.40) ^a^	0.031 (<0.012–0.078) ^a^	[102]
China	8235	0.16 ^b^ (0.02–0.57) ^a^	N.A.	[100]
Sweden	227	0.41	N.A.	[66]
Italy	108	0.37	N.A.	[70]
Italy	123	N.A. (0.08–0.95)	N.A.	[103]
Italy	22	0.44 (0.19–0.73)	0.022 (0.010–0.048)	[104]
Italy	26	0.56 (0.17–0.98)	N.A.	[105]
Japan	53	0.33	Not detected	[106]
Japan	55	1.47	Not detected	[107]
Germany	12	0.45 (0.12–0.78)	N.A.	[57]

^a^ 5th–95th percentiles; ^b^ median; N.A., not available.

**Table 4:** Hexane losses in the oilseed crushing industry.

Sources of Hexane Losses	Percentage of Total Losses	Kg Hexane Lost per Ton of Seeds
Meals	30–60%	0.05–0.3
Crude oil	2–6%	0.02–0.05
Exhaust from mineral oil system and drying/cooling vents	5–20%	0.05–0.15
Fugitive losses and losses from start-ups and shutdowns	14–63% ^a^	0.1–0.5
Wastewater	Negligible	Negligible

^a^ Calculated as a balance of previous losses.

**Table 5:** *n*-Hexane residues in food and health food products.

Product	Type of Product	Highest Detected *n*-Hexane Concentration (mg/kg)	LOD; LOQ (mg/kg)	Analytical Method ^a^	*n*-Hexane Concentration > LOQ/Total Analysed Products	Products Origin Country	References
Refined oil	Frying oil	0.009	0.003; 0.005	SPME-GC-FID	14/40	Iran	[108]
	Blended oil	0.010					
	Sunflower oil	0.005					
	Corn oil	0.018					
	Canola oil	0.043					
	Orujo oil	3	0.7; 2.3	HS-MS	2/50	Spain	[109]
	Primrose oil	0.70	0.004; 0.125	HS-GC-FID	3/14	Poland	[110]
	Soybean oil	1.4	N.A.; 0.5	HS-GC-FID	8/87	Korea	[111]
	Sesame oil	0.9					
	Chinese pepper oil	1.2					
	Rice-bran oil	0.7					
	Corn oil Margarine	0.6 2.8					
	Olive oil	0.400	0.003; 0.005	SPME-GC-FID	12/16	N.A.	[112]
	Rape seed oil	0.233					
	Sunflower oil	0.005					
	Soybean oil	0.055					
	Pumpkin oil	0.056					
	Grape seed oil	0.011					
	Commercial frying oil	11.10	1.20; 5.05	HS-SPME-GC-FID	6/10	Iran	[113]
	Semi-solid oil	10.00					
	Sunflower oil	2.688	N.A.; 0.25	HS-GC-FID	3/4	Malaysia	[114]
	Corn oil	0.358					
	Sesame oil	0.602					
	Palm oil	0.126					
Commercial foods and health food products	ω -3 Fatty acid containing oils	0.66	0.16; 0.47	HS-GC-MS	N.A./60	Korea	[115]
	γ-Linolenic acid containing oils	0.44					
	Conjugated linoleic acid	1.09					
	Lecithin	0.85					
	Octacosanol containing oils	0.54					
	Lutein	0.50					
	Extract of Oenothera biennis	0.26					
	Coenzyme Q-10	0.12					
	Phosphatidylserine	0.81					
	Commercial products containing annatto extracts as colourant	0.7	0.01; 0.30	HS-GC-FID	2/23	Japan	[116]

^a^ SPME, solid phase micro-extraction; GC, gas chromatography; FID, flame-ionisation detector; HS, headspace sampler; MS, mass detector; N.A., not available.

## Data Availability

Not applicable.
