# Correction: Cravotto et al. Towards Substitution of Hexane as Extraction Solvent of Food Products and Ingredients with No Regrets. *Foods* 2022, *11*, 3412

**DOI:** 10.3390/foods15193560

**Published:** 2026-10-08

**Authors:** Christian Cravotto, Anne-Sylvie Fabiano-Tixier, Ombéline Claux, Maryline Abert-Vian, Silvia Tabasso, Giancarlo Cravotto, Farid Chemat

**Affiliations:** 1 GREEN Extraction Team, INRAE, UMR 408, Avignon University, F-84000 Avignon, France; christian.cravotto@alumni.univ-avignon.fr (C.C.); anne-sylvie.fabiano@univ-avignon.fr (A.-S.F.-T.); ombeline.claux@alumni.univ-avignon.fr (O.C.); maryline.vian@univ-avignon.fr (M.A.-V.); 2 Department of Drug Science and Technology, University of Turin, Via P. Giuria 9, 10125 Turin, Italy; silvia.tabasso@unito.it

## Text Correction

There was an error in the original publication [1]. The potential conflict of interest was not clearly disclosed.

A correction has been made to “Conflicts of Interest”

“Author Prof. Farid Chemat and Prof. Anne Sylvie Fabiano Tixier were co-inventors of patent FR3090008B1, originally filed by Avignon Université and Pennakem Europa in December 2018. However, Avignon Université transferred its rights related to this patent to Pennakem Europa in December 2019, before the submission and publication of the article. Therefore, at the time of manuscript submission and publication, the academic authors did not hold ownership rights or active financial interests related to this patent. Author Dr. Ombéline Claux contributed to the manuscript while conducting her PhD research at Avignon Université, where the scientific work underlying the review was prepared. Although she was employed by Pennakem Europa at the time of publication, her substantial contribution to the manuscript had already been completed in an academic context. Therefore, the affiliation reported in the article reflected the institution where her contribution was carried out. The remaining authors declare that the research was conducted in the absence of any commercial or financial relationships that could be construed as potential conflicts of interest.”

The authors state that the scientific conclusions are unaffected. This correction was approved by the Academic Editor. The original publication has also been updated.
